# Community Detection on Networks with Ricci Flow

**DOI:** 10.1038/s41598-019-46380-9

**Published:** 2019-07-10

**Authors:** Chien-Chun Ni, Yu-Yao Lin, Feng Luo, Jie Gao

**Affiliations:** 1 Yahoo! Research, Sunnyvale, CA USA; 2 Intel Inc., Hillsboro, OR USA; 3 Rugters University, New Brunswick, NJ USA; 4 Stony Brook University, Stony Brook, NY USA

**Keywords:** Applied mathematics, Computational science, Computer science

## Abstract

Many complex networks in the real world have community structures – groups of well-connected nodes with important functional roles. It has been well recognized that the identification of communities bears numerous practical applications. While existing approaches mainly apply statistical or graph theoretical/combinatorial methods for community detection, in this paper, we present a novel geometric approach which  enables us to borrow powerful classical geometric methods and properties. By considering networks as geometric objects and communities in a network as a geometric decomposition, we apply curvature and discrete Ricci flow, which have been used to decompose smooth manifolds with astonishing successes in mathematics, to break down communities in networks. We  tested our method on networks with ground-truth community structures, and experimentally confirmed the effectiveness of this geometric approach.

## Introduction

Complex networks have been used to model connections of elements in many different fields such as social networks, biology, and biochemistry (protein-protein networks^1^, metabolic networks, and gene networks), and computer science (P2P, the Internet). It has been widely recognized that many real world networks have community structures – nodes in the same community are densely connected while nodes from different communities are sparsely connected. Recognition of community structures brings out important functional components and plays an important role in supporting processes on networks such as contagions of diseases, information or behaviors. Many algorithms have been developed to identify and separate communities in the literature^2–11^.

Most current work on community detection try to recognize dense clusters in a graph: by randomized algorithms such as label propagation^12^ or random walks^13^; by optimized centrality such as betweenness centrality^14^; or by considering notions such as modularity^15^: the fraction of edges that fall within the given groups minus the expected fraction if edges were distributed uniformly at random (while still respecting the degree distribution). The viewpoint of modularity could be considered as a statistical measure of non-uniformity of the network.

Unlike existing methods, our work explores a new path connecting community detection and geometry. We consider community structure as a geometric phenomenon and use geometric methods to identify communities in a network. The motivation comes from the classical topological connected sum decomposition of 3-manifolds. The groundbreaking work of Hamilton and Perelman^16,17^ shows that the connected sum decomposition can be detected by the geometric Ricci flow. By considering a network as a discrete counterpart of a manifold and connected sum components as communities, we introduce a discrete Ricci flow on networks for identifying communities in a network.

The Ricci flow approach is based on the geometric notion of curvature, introduced by F. Gauss and B. Riemann over 150 years ago, which describes quantitatively how spaces are bent at each point^18^. In classical geometry, regions in a space with large positive curvature tend to be more densely packed than regions of negative curvature. To locate these regions of large curvature, in a seminal work in 1982, Hamilton^16^ introduced a curvature guided diffusion process, called the Ricci flow, that deforms the space in a way formally analogous to the diffusion of heat. Under the Ricci flow, regions in a space of large positive curvature shrink to points whereas regions of very negative curvature spread out. In this paper, we observed that communities in networks resemble regions in Riemannian manifolds of large positive curvature. By applying the discrete Ricci flow on networks as the classic Ricci flow on manifolds, we are able to detect community structures in networks.

**Figure 1:**
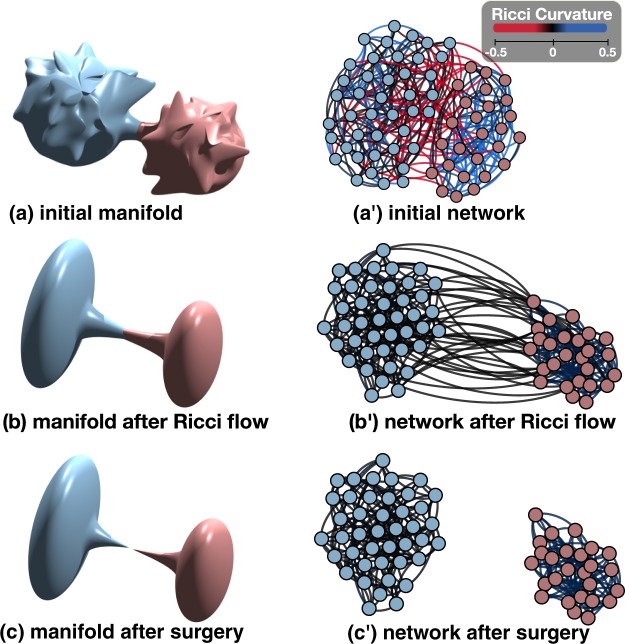
An illustration of Ricci flow on a manifold and a network. Ricci flow captures the large positive curvature regions in the manifold as well as communities in the network. The formation of singularities in the Ricci flow is illustrated by (**b,c**). The analog in (**b’,c’**) in the discrete Ricci flow decomposes the network into communities.

Figure 1 illustrates this key observation. In the left column, the Ricci flow deforms a Riemannian manifold (Fig. 1(a)) gradually and develops a neck pinching singularity (Fig. 1(b)). By removing the singularity, the manifold is decomposed into sub-regions of positive curvature (Fig. 1(c)). In the right column, the discrete Ricci flow on a metric graph (Fig. 1(a’)) stretches edges of large negative Ricci curvature and shrinks edges of large positive Ricci curvature over time (Fig. 1(b’)). By removing the edges of length greater than a threshold value, we recover subgraphs of large Ricci curvature representing communities (Fig. 1(c’)).

### Our contribution

To carry out the discretization process, we start from the recent important work of Y. Ollivier^19–21^ which introduced Ricci curvature on metric spaces by using the optimal transport theory. Ollivier’s definition for metric graphs assumes a probability measure for each node and the Ricci curvature of an edge is  related to the optimal transportation cost between two probability measures defined on the vertices of the edge. Various definitions of Ricci curvatures on networks have been used in graph analysis for applications such as anomaly detection, detection of backbone edges or cancer related proteins^22–30^.

Motivated by Hamilton’s Ricci flow, we introduce an algorithm, called discrete Ricci flow on networks, for detecting community structures. The discrete Ricci flow is defined on weighted graphs and deforms edge weights as time progresses: edges of large positive Ricci curvature (i.e., sparsely traveled edges) will shrink and edges of very negative Ricci curvature (i.e., heavily traveled edges) will be stretched. By iterating the Ricci flow process, we are able to identify heavily traveled edges and thus find communities.

**Figure 2:**
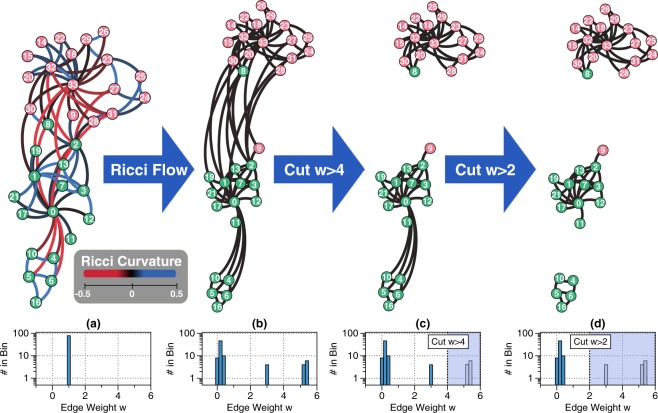
Ricci flow for community detection on the Karate club graph generated by Gephi’s ForceAtlas2 layout^59^. (**a**) The Karate club graph with edge weight 1 on all edges. Different colors of vertices represent different communities. The colors of edges represent the Ricci curvature on the edges. Notice that most edges between communities are negatively curved. (**b**) The same graph after 100 Ricci flow iterations. Ricci flow adjusts the edge weights so that the edge Ricci curvatures are the same everywhere; the intra-community edges shrink; the inter-community edges are stretched. (**c**) By removing all edges with weight greater than 4, we acquire a partitioning of the graph with two communities. (**d**) By removing all edges with weight greater than 2, we obtain three communities.

**Figure 3:**
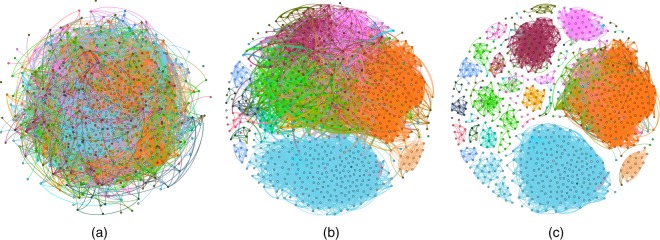
(**a**) A Facebook ego network of one user with 792 friends and 14025 edges generated by Gephi’s Fruchterman Reingold layout^59^. The colors represent 24 different friend circles (communities) hand labeled by the user. (**b**) By the Ricci flow process of 20 iterations, the weights of inter-community edges are increased (thick edges in the figure) while the weights of intra-community edges gradually shrink to 0 (thin edges in the figure). (**c**) By removing the inter-community edges with high weights, the communities are clearly detected.

Figure 2 illustrates how discrete Ricci flow detects communities on the Zachary’s Karate club graph. In this graph, individuals in the same club are represented as nodes of the same color, and friendship ties between two individuals are represented as edges with weights equal to 1. With discrete Ricci flow algorithm, the edge weights evolve. By this, the community structure can be easily detected by removing edges that are stretched greater than a threshold. Figure 3 shows another example of communities on a Facebook ego graph. We have also tested our Ricci flow algorithms on many of the real-world networks with ground-truth communities and artificial networks, and shown competitive accuracy results with other community detection algorithms using various statistical methods or physics models.

We applied the discrete Ricci flow method on artificial networks generated by the stochastic block model (SBM)^11^, the Lancichinetti-Fortunato-Radicchi (LFR) benchmark graph^31^ and the emergent geometrical network model^32,33^ (GNet). We choose Adjusted Rand Index (ARI)^34^ as a quality measure for the clustering accuracy. The proposed discrete Ricci flow method is shown to provide nearly perfect clustering result when community structures exist. Also, extensive comparison tests on real networks with ground-truth communities show that our algorithm is competitive with previously proposed ones. Similar results have been observed with other metrics of clustering accuracy such as modularity.

Our work of Ricci curvature on networks is built on our previous work^22,30^ and is also inspired by the important works of E. Saucan and J. Jost *et al*. in^23,27,35,36^. In these works, they systematically introduced and investigated various discrete curvatures for complex networks. The comparative analysis of Forman and Ollivier Ricci curvature on benchmark datasets of complex networks and real-world networks was also carried out. Their numerical results show a striking fact that these two completely different discretizations of the Ricci curvatures are highly correlated in many networks.

### Related work

Ricci curvature on general spaces without Riemannian structures has been recently studied, in the work of Ollivier^19,20^ on Markov chains, and Bakry and Emery^37^, Lott, Villani^21^, Bonciocat and Sturm^38,39^ on general metric spaces. Ricci curvature based on optimal transportation theory, proposed by Ollivier (Ollivier-Ricci curvature)^19,20^, has become a popular topic and has been applied in various fields – for distinguishing cancer-related genes from normal genes^28^, for studying financial market fragility^29^, for understanding phylogenetic trees^26^, and for detecting network backbone and congestion^22,25,40^. In^41^, Pal *et al*. proposed to use Jaccard coefficients for a proxy for Ollivier-Ricci Curvature. Besides, discrete Ricci curvature has also been defined on cell complexes, proposed by Forman^42^ (Forman curvature or Forman-Ricci curvature). Forman curvature is based on graph Laplacian. It is easier and faster to compute than Ollivier-Ricci curvature, but is less geometrical. It is more suitable for large scale network analysis^23,24,43,44^ and image processing^45^. We have also experimented with Forman curvature for community detection. The results were less satisfying. So here we focus on Ollivier Ricci curvature.

Unlike discrete Ricci curvature, discrete Ricci flow has not been studied as much. Chow and Luo introduced the first discrete Ricci flow on surfaces^46^. In^43^, Weber *et al*. suggested applying Forman-Ricci flow for anomaly detection in the complex network. In^30^, Ni *et al*. used the Ollivier-Ricci curvature flow to compute the Ricci flow metric as edge weights for the problem of network alignment (noisy graph matching).

Community detection, on the other hand, is a well-studied topic in social network analysis^2,3,6,47–51^, and protein-protein interaction networks^1,52^. There are a few main ideas. One family of algorithms iteratively remove edges of high ‘centrality’, for example, the edge betweenness centrality as suggested in^14^ by Girvan and Newman. The other idea is to use modularity (introduced by Newman and Clauset *et al*.), which measures the strength of division of a graph into clusters^4,7^, as the objective of optimization. But methods using modularity suffer from a resolution limit and cannot detect small communities. A geometric extension, named Laplacian modularity, is also suggested with the help of Gauss’s law in^5^. Another family of algorithms borrows intuitions from other fields. In^53^, a spin glass approach uses the Potts model from statistical physics: every node (particle) is assigned one of *c* spin states (communities); edges between nodes model the interaction of the particles. The community structure of the network is understood as the spin configuration that minimizes the energy of the spin glass. In^12^, Raghavan *et al*. proposed a non-deterministic label propagation algorithm for large networks. In the initial stage, the algorithm randomly assigns each node in the graph one of *c* labels. Each node then changes its label to the most popular label among its neighbors. Infomap^13^ uses an information theoretic approach. A group of nodes for which information flows quickly shall be in the same community. The information flow is approximated by random walks and succinctly summarized by network coding.

Taking a geometric view of complex networks is an emerging trend, as shown in a number of recent work. For example, the community structures were used as a coarse version of its embedding in a hidden space with hyperbolic geometry^54^. Topological data analysis, a typical geometric approach for data analysis, has been applied for analyzing complex systems^55^.

## Classical Theory of Ricci Curvature, Optimal Transport and the Ricci Flow

In this section, we briefly recall the basic notation of Ricci curvature in Riemannian geometry, Ollivier’s work on generalizing Ricci curvature to metric measured spaces through optimal transport, and the Ricci flow. Their discrete and computational counterparts are addressed in Section 3.

### Sectional and Ricci curvature

**Figure 4:**
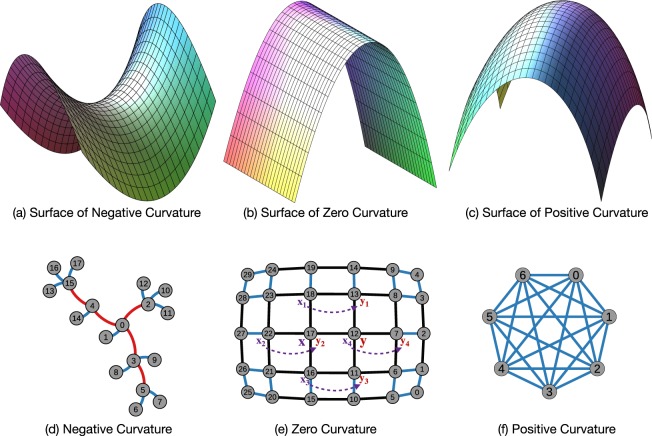
Examples of Ricci curvature on manifolds and graphs. In (**a**–**c**), manifolds with negative, zero, and positive curvatures are shown. In (**d**–**f**), all edges have weight of 1. (**f**) A complete graph with all edges of positive curvature. (**e**) A (infinitely sized) grid graph with all edges of zero curvature. The cost of moving *m*_*x*_ = {*x*, *x*_1_, *x*_2_, *x*_3_, *x*_4_} to *m*_*y*_ = {*y*, *y*_1_, *y*_2_, *y*_3_, *y*_4_} is equal to *d*(*x*, *y*). (**d**) A tree graph with negative curvature everywhere, except the edges of the leaves.

One of the central themes in modern geometry is the notion of curvature which quantitatively measures how space is curved. It was introduced by Gauss and Riemann. For a surface in the 3-dimensional Euclidean space, the *Gaussian curvature* at a point is defined as the signed area distortion of the Gauss map sending a point on the surface to its unit normal vector. For instance, a plane has zero curvature, a sphere has positive curvature and a hyperboloid of one sheet has negative curvature (Fig. 4). Gauss showed that curvatures depend only on the induced Riemannian metric on the surface, i.e., independent of how a surface is embedded in the 3-dimensional space.

For a Riemannian manifold (*M*, *g*), Riemann’s *sectional curvature* assigns a scalar for each 2-dimensional linear subspace *P* in the tangent space at a point *p* of *M*. This scalar is equal to the Gaussian curvature of the image of *P* under the exponential map at *p*. A positive sectional curvature space tends to have a small diameter and is geometrically crowded (e.g., a sphere). In contrast, a negative sectional curvature closed Riemannian manifold has an infinite fundamental group, a contractible universal cover, and is geometrically spreading out like a tree in large scale. Thus, a positively curved region behaves more like a “community” than negatively curved regions. Similar to sectional curvature, the *Ricci curvature* assigns each unit tangent vector *v* at *p* a scalar which is the average of the sectional curvatures of planes containing *v*. Geometrically, Ricci curvature controls how fast the volume of a ball grows as a function of the radius. It also controls the volume of the overlap of two balls in terms of their radii and the distance between their centers. On the other hand, the volume of the overlap of two balls is directly related to the cost of transportation to move one ball to the other, i.e., a larger volume of overlap means less cost of moving one ball to the other. It shows that the Ricci curvature is related to optimal transportation. An explicit formula (Equation 1) that builds a bridge between them was worked out by Ollivier^19^. Through the formula, Ollivier defined the generalized Ricci curvature on metric measure spaces by the optimal transportation.

### The optimal transportation and ollivier’s ricci curvature

The original optimal transport problem was proposed by G. Monge in 1781. The problem wants to minimize the transportation cost to move iron ores from different mines to a collection of factories which consume the iron ores. In Monge’s setting, the problem can be mathematically formulated as follows. Let mines and factories as two probability spaces *X* and *Y*; the amount of iron ores to be moved and consumed as two probability Borel measures *μ* and *ν*, we define the cost of transporting from location *x* to location *y* to be *c*(*x*, *y*), where $c:X\times Y\to {{\mathbb{R}}}_{\ge 0}$. In general, the cost function *c* is usually taken to be the distance *d*(*x*, *y*) if *X* = *Y* and the cost of transportation per-unit distance is constant. A *transportation T*: (*X*, *μ*) → (*Y*, *ν*) is a measure preserving map. Monge’s formulation of the optimal transportation problem is to find a transportation *T*: *X* → *Y* that realizes ${\rm{\inf }}\{\mathop{\int }\limits_{X}\,c(x,T(x))\,{\rm{d}}\mu (x)\,|{\rm{T}}:{\rm{transportation}}\}$.

Monge’s optimal transportation problem had a major breakthrough in 1930 when Kantorovich formulated the optimal transportation problem into a linear optimization problem. In his setting, Kantorovich replaces transportation maps *T* by probability measures *γ* on *X* × *Y* (called *transportation plans*) satisfying *γ*(*A* × *Y*) = *μ*(*A*) and *γ*(*X* × *B*) = *ν*(*B*) for all measurable subsets *A* and *B*. The goal is to find a transportation plan *γ* that attains the infimum cost

$$W(\mu ,\nu )={\rm{\inf }}\{\mathop{\int }\limits_{X\times Y}\,c(x,y)\,{\rm{d}}\gamma (x,y)|\gamma \in {\rm{\Gamma }}(\mu ,\nu )\},$$

where Γ(*μ*, *ν*) denotes the collection of all possible transportation plans. If *X* is a metric space with distance function *d* and *X* = *Y*, the quantity *W*(*μ*, *ν*) for *c*(*x*, *y*) = *d*(*x*, *y*) is called the *Wasserstein distance* (or the earth mover’s distance) between two probability measures *μ*, *ν* on *X*.

Wasserstein distance plays a crucial role in Ollivier’s approach to Ricci curvature. In his observation^19^, if (*M*^*n*^, *d*) is an n-dimensional Riemannian manifold with Riemannian volume *μ* and fix *ε* > 0, let ${m}_{x}=\frac{\mu {|}_{B(x,\varepsilon )}}{\mu (B(x,\varepsilon ))}$ be the probability measure associated to *x* ∈ *M* where *B*(*x*, *ε*) is the ball of radius *ε* at *x*. Then the Wasserstein distance *W*(*m*_*x*_, *m*_*y*_) = (1 − *k*(*x*, *y*))*d*(*x*, *y*), where

1 $$k(x,y)=\frac{{\varepsilon }^{2}{\rm{Ricci}}(v,v)}{2(n+2)}+O({\varepsilon }^{3}+{\varepsilon }^{2}d(x,y))$$

and *v* is the tangent vector at *x* of the geodesic *xy*. This shows that Ricci curvature can be defined for general metric spaces with measures. Given a metric space (*X*, *d*) equipped with a probability measure *m*_*x*_ for each *x* ∈ *X*, the Ollivier’s Ricci curvature along the path *xy* is defined to be

2 $${\kappa }_{xy}=1-\frac{W({m}_{x},{m}_{y})}{d(x,y)},$$

where *W*(*m*_*x*_, *m*_*y*_) is the Wasserstein distance with respect to *c*(*x*, *y*) = *d*(*x*, *y*).

### The Ricci flow

The Ricci flow, introduced by Richard S. Hamilton in 1981^16^, deforms the metric of a Riemannian manifold in a way formally analogous to the diffusion of heat, smoothing out irregularities in the metric. The Ricci flow has been one of the most powerful tools for solving geometric problems in the past forty years. The flow exhibits many similarities with the heat equation.

Suppose a Riemannian metric *g*_*ij*_ is given on a manifold *M* so that its Ricci curvature is *R*_*ij*_. Hamilton’s Ricci flow is the following second-order nonlinear partial differential equation on symmetric (0, 2)-tensors:

$$\frac{\partial }{\partial t}{g}_{ij}=-\,2{R}_{ij}.$$

A solution to the Ricci flow is a one-parameter family of metrics *g*_*ij*_(*t*) on a smooth manifold *M* satisfying the above partial differential equation. One of the key properties of the Ricci flow is that the curvature evolves according to a nonlinear version of the heat equation. Thus the Ricci flow tends to smooth out irregularity of the curvature. Under the Ricci flow, regions in the manifold of positive sectional curvature tend to shrink and regions of negative sectional curvature tend to expand and spread out. Singularities usually occur while deforming a Riemannian 3-manifold through the Ricci flow. They appear in a small neighborhood of a surface in the 3-manifold. By removing the singularities (i.e., surfaces) and redefining the Ricci flow on the remaining pieces, one produces the Ricci flow with surgery on the manifold. Figure 1(b,c) illustrate the formation of a singularity and the ‘surgery’ operation. The ground-breaking work of Perelman^17^ shows that the Ricci flow with surgery captures the geometric decomposition of the 3-manifold. It solves the Geometrization Conjecture of Thurston and geometrically classifies all 3-manifolds.

Ricci flow enables a better understanding of the evolution and community structure of networks. In our heuristic thinking, a network is analogous to a discretization of high dimensional manifold (say a 3-manifold) and communities in the network are analogous to the components in the geometric decomposition of the 3-manifold. Since Perelman’s work^17^ proved that the Ricci flow is able to predict geometric components of a 3-manifold, it suggests that a discrete Ricci flow on the network should be able to detect the community structure. Just like in Hamilton-Perelman’s work on Ricci flow, the cutoff number of iterations and threshold value for surgery in Ricci flow depend on individual networks.

## Theory and Algorithms on Discrete Ollivier Ricci Curvature Flow

In this section, we introduce our discrete Ricci flow algorithm for community detection on the network. We started with the definition of Ricci curvature by Ollivier in Equation 2, for each node x on a metric graph *G* = (*V*, *E*, *w*), we define a mass distribution *m*_*x*_ on *x*’s neighbor nodes. A discrete *transport plan* is a map *A*: *V* × *V* → [0, 1] such that *A*(*u*, *v*) is the amount of mass at vertex *v* to be moved to vertex *u*. It satisfies $\sum _{v^{\prime} \in V}\,A(u,v^{\prime} )={m}_{x}(u)$ and $\sum _{u\text{'}\in V}\,A(u^{\prime} ,v)={m}_{y}(v)$. The Wasserstein distance here *W*(*m*_*x*_, *m*_*y*_) is defined as the minimum total weighted travel distance to move *m*_*x*_ to *m*_*y*_, i.e., $W({m}_{x},{m}_{y})={\rm{\inf }}\{\sum _{u,v\in V}\,A(u,v)d(u,v)\}$. The discrete Ricci curvature on a network edge *xy* ∈ *E* is defined as

$${\kappa }_{xy}=1-\frac{W({m}_{x},{m}_{y})}{d(x,y)},$$

where *d*(*x*, *y*) is the length of the shortest path between nodes *x* and *y*.

Under this definition, if two nodes *x* and *y* are from different communities, their neighbor nodes tend to have fewer common neighbors, hence the best way to move *m*_*x*_ from *x*’s neighbors to *m*_*y*_ in *y*’s neighbors is to travel along the edge *xy*. Because of this, the Wasserstein distance is necessarily larger than the length of *xy*, which leads to negative Ricci curvature. Alternatively, nodes within the same community tend to share neighbors or have shortcut between neighbors, thus have a Wasserstein distance no greater than *d*(*x*, *y*). Therefore intra-community edges are mostly positively curved. See Fig. 4 for examples of network edges of positive, zero and negative curvatures.

Note that the probability distribution *m*_*x*_ for *x* ∈ *V* needs to be specified. In previous work^56^, the probability distribution is uniform on *x’s* neighbors. In this paper, we suggest a more general family of probability distributions ${m}_{x}^{\alpha ,p}$, with two parameters: *α* ∈ [0, 1] and power *p* ≥ 0:

$${m}_{x}^{\alpha ,p}({x}_{i})=(\begin{array}{ll}\alpha  & {\rm{if}}\,{x}_{i}=x\\ \frac{1-\alpha }{C}\cdot \exp (\,-\,d{(x,{x}_{i})}^{p}) & {\rm{if}}\,{x}_{i}\in \pi (x)\\ 0 & \mathrm{otherwise}.\end{array}$$

Here $C=\sum _{{x}_{i}\in \pi (x)}\,\exp (\,-\,d{(x,{x}_{i})}^{p})$ is a normalization factor and *π*(*x*) is the set of neighbors of *x*. The parameter *α* determines the probability to remain at *x*. The power parameter *p* determines how much we want to discount the neighbor *x*_*i*_ of *x* with respect to the weight *d*(*x*, *x*_*i*_). When *p* = 0, the probability measure is uniform on all neighbors of *x* as suggest in^56^. For a large *p*, the neighbors that are far away from *x* are aggressively discounted.

The discrete Ricci flow algorithm on a network is an evolving process. In each iteration, we update all edge weights simultaneously by the following flow process:

$${w}_{xy}^{(i+1)}={d}^{(i)}(x,y)-{\kappa }_{xy}^{(i)}\cdot {d}^{(i)}(x,y),$$

where ${w}_{xy}^{(i)}$ is the weight of the edge *xy* at the *i*-th iteration, and ${\kappa }_{xy}^{(i)}$ is the Ricci curvature at the edge *xy* at the *i*-th iteration, and *d*^(*i*)^(*x*, *y*) is the shortest path distance on the graph induced by the weights ${w}_{xy}^{(i)}$. Initially ${w}_{xy}^{(0)}={w}_{xy}$ and ${d}_{xy}^{(0)}={d}_{xy}$. The detailed algorithm is presented in Supplementary Information.

This discrete Ricci flow process expands negatively curved edges and shrinks positively curved edges. Eventually, nodes connected by intra-community edges are condensed and inter-community edges are stretched. By this effect, a simple thresholding procedure can easily separate different communities. This is termed network ‘surgery’ when edges of large weights (likely inter-community edges) are removed after several Ricci flow iterations (usually 10 to 15 iterations). See Fig. 2 as an example for the surgery process. For networks with hierarchical community structures, we may perform multiple rounds of network surgery and Ricci flow to fully separate communities at different scales.

## Results

### Theoretical results

We can prove rigorously that the Ollivier Ricci flow with respect to the specific choice of *α* = 0 and *p* = 0 can successfully detect community structure for the following *G*(*a*, *b*) family of graphs (Please refer to Supplementary Information for further detail). Take the complete graph on *b* + 1 vertices *p*_1_, ..., *p*_*b* + 1_ and *b* + 1 complete graphs *C*_1_, ..., *C*_*b* + 1_ on *a* + 1 vertices. Take a vertex *u*_*i*_ from each *C*_*i*_ and identify *u*_*i*_ with *p*_*i*_. The resulting graph is *G*(*a*, *b*). For *a* > *b*, this is a highly symmetric graph with a clear community structure – each copy of *C*_*i*_ is a community and there are *b* + 1 of them. Between any two communities *C*_*i*_, *C*_*j*_, there is only one edge *u*_*i*_*u*_*j*_ joining them. This community structure can be detected by the Ollivier Ricci flow with respect to the Ollivier Ricci curvature *K*_0_ corresponding to *α* = 0, *p* = 0 in Section 3. More precisely, the Ollivier Ricci curvature *K*_0_ is associated with the probability distribution *μ*_*x*_ such that *μ*_*x*_(*y*) = 1/*d*_*x*_ if *y* is adjacent to *x* and *μ*_*x*_(*y*) = 0 otherwise. In this case, we are able to compute explicitly the Ollivier Ricci curvature at the *n*-th iteration of the Ricci flow and confirm how the weights of the network edges evolve over time.

**Theorem 4.1**. *The Ricci flow associated to the Olivier K*_0_*-Ricci curvature detects the community structure on G*(*a*, *b*) *if a* > *b* ≥ 2, *namely, the weight of the intra-community edges shrink asymptotically faster than the weight of the inter-community edges*.

**Proof**. Please refer to Supplementary Information.

### Experimental results

In this section, we explain the model networks and real-world datasets used to evaluate the community detection accuracy of our method. For the model network, we tested the growing geometrical network model with emergent complex geometry (GNet), and two models that provides community labels: the standard and widely used stochastic block model (SBM), and the Lancichinetti-Fortunato-Radicch benchmark model (LFR) that generates graphs of power-law degree distributions. For real-world datasets, we picked 6 different community graphs that come with *ground-truth community* labels. More detailed experiments can be found in Supplementary Information.

#### Model networks and real world datasets

Stochastic Block Model: The *stochastic block model* (SBM) is a probabilistic graph model^11^. A graph following the stochastic block model has *n* vertices, which are partitioned into *k* communities. Two nodes within a community are connected with probability *p*_*intra*_ while two nodes in different communities are connected with probability *p*_*inter*_, *p*_*intra*_ > *p*_*inter*_.

Lancichinetti-Fortunato-Radicch Model: The Lancichinetti-Fortunato-Radicch (LFR) benchmark^31^ generates undirected unweighted networks with non-overlapping communities. The model produces networks with both degree and community size satisfying power-law distributions. This model is also commonly used to evaluate community detection algorithms^2^.

Emergent Geometrical Network Model: The emergent geometrical network model^32,33^ (GNet) describes a growing network with a high clustering coefficient using the triadic closure property. It is observed to have non-trivial community structures. One version described in^33^ could grow a geometric network. It is composed of the skeleton of a simplicial complex in which a set of 2-simplices are glued together properly. The generation of this model is controlled by the designated number *m* of 2-simplices glued along a 1-simplex (edge), and the probability *p* of connecting two nodes with hop distance 2.

Real World Datasets: For real world datasets, we choose networks that provide ground truth communities from KONECT^57^, UCI network data repository and Stanford Network Analysis Project^58^. The statistics of the real world datasets are summarized in Table 1. In the followings, we briefly describe the datasets.

- *Karate club network*. The Karate club network data set was collected from the members of a university karate club by Wayne Zachary in 1970s. The network is undirected in which nodes represent members of the club, and edges represent ties between two members. This data set is generally used to find the two groups of people into which the karate club fission after a conflict between two faculties.
- *American college football network*. The American college football network is a representation of the schedule of Division I games during the season Fall 2000 and was previously used for community detection by Girvan and Newman. Each node represents a football team and each edge indicates a game between two teams. The community structure of the network is given by partitioning the teams into 12 conferences. Games held between teams of the same conference are held more frequently than games played between different conferences.
- *Political books network*. This is a network of books about US politics published around the time of the 2004 presidential election and sold by the online bookseller *Amazon.com*. Edges between books represent frequent co-purchasing of books by the same buyers.
- *Political blogs network*. The 2004 U.S. Presidential Election was notably influenced by blogs. The political blogs network data set was collected by Adamic and Glance in 2005. The posts published by either liberal or conservative bloggers are represented by nodes. Any two nodes are connected by an edge if one of them is cited by the other.
- *Ego-network from Facebook*. The ego-network dataset consists of ‘friend circles’ of one anonymous user and his/her friends on Facebook. The network forms friend circles such as family members, high school friends or other friends that are ‘hand labeled’ by the user. To normalize the influence of users belongs to multiple circles, we treat the overlaid circle as a new circle.
- *Email-EU-core network*. The Email-Eu-core network was formed by the email contacts between members of a large European research institution. The members are represented by nodes where any pair of nodes are connected by an edge if they have had contacts through e-mail. Each individual belongs to exactly one of 42 departments at the research institute.

**Table 1:** Real World Datasets.

Datasets	V	E	#Class	AvgDeg	CC	Diameter
Karate Club	34	78	2	4.5882	0.5706	5
Football	115	613	12	10.6608	0.4032	4
Polbooks	105	441	3	8.4000	0.4875	7
Polblogs	1222	16714	2	27.3552	0.3203	8
FB-Ego	792	14025	24	35.417	0.483	10
Email-EU-core	1005	16064	42	31.968	0.450	7

#### Experimental results

To evaluate the clustering accuracy of our algorithm, we tested the clustering result with two different metrics: Adjusted Rand Index (ARI) and modularity. ARI measures the accuracy of clustering result with the *ground truth* clustering. Modularity quantifies the strength of the community structure of a given graph without the need of ground-truth clustering.

**Figure 5:**
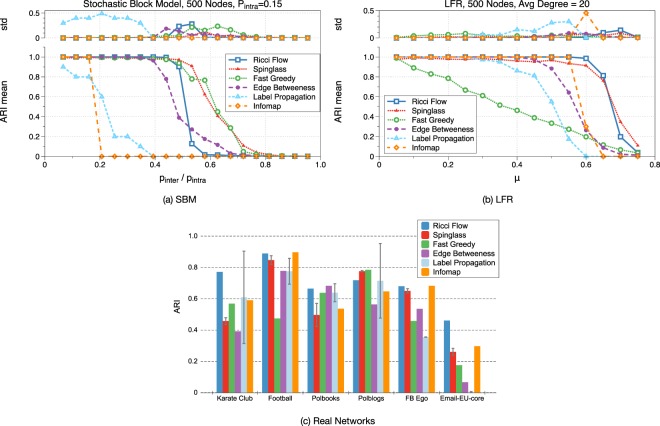
The accuracy of the Ricci flow method for community detection on model networks. The accuracy is measured by the adjust Rand index (ARI) and each data point is the average of 10 model graphs. In (**a**), we tested on the stochastic block model (SBM) with 500 nodes and two communities of the same size. A graph with low *p*_*inter*_/*p*_*intra*_ ratio has more distinctive communities. Our method is shown to have perfect accuracy with *p*_*inter*_/*p*_*intra*_ < 0.5. In (**b**), for Lancichinetti-Fortunato-Radicchi (LFR) Model, we set the graph to have 500 nodes, average degree of 20, with 38 communities. LFR can produce graphs with power-law degree distribution with communities of different sizes. The magnitude of community structure is controlled by *μ*, the ratio of inter-community edges with intra-community edges. Again, our method produces the best accuracy among all methods. In (**c**), for non-deterministic algorithm Spinglass and Label Propagation, the accuracy are averaged over 10 runs.

Clustering Accuracies: The Clustering accuracies of applying discrete Ricci flow for 50 iterations is shown in Fig. 5. In Fig. 5(a,b), the parameters *p*_*inter*_/*p*_*intra*_ of the SBM and *μ* of the LFR indicate the magnitude of community structure of the models respectively. In both models, higher parameter values in *x*-axis indicate weaker community structures. We choose the adjusted Rand index (ARI)^34^ as the quality measure for the clustering accuracy compared with the ground truth, as shown in the vertical axes. ARI scores the agreement of partitioned node pairs in ground truth communities and clustered communities. The higher ARI score is, the more accurate our detected communities are. The results of Ricci flow algorithm show robust detection of community structures that compares favorably with prior algorithms – with a sharp phase transition from nearly 100% accuracy for SBM models with *p*_*inter*_/*p*_*intra*_ = 0.5 (almost all nodes separated correctly) to nearly 0% accuracy for models with *p*_*inter*_/*p*_*intra*_ = 0.55 (meaning the non-existence of community structure). Similar results have been observed with modularity.

**Figure 6:**
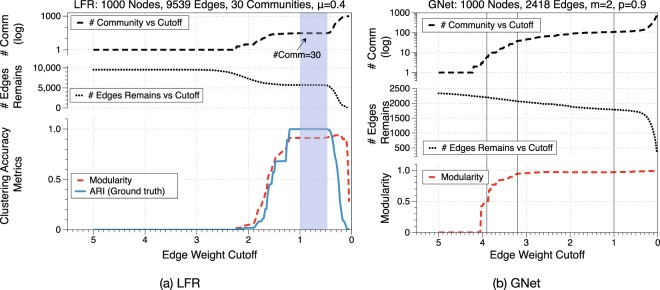
A comparison of clustering accuracy on an LFR graph after 50 iterations and GNet after 20 iterations of the Ricci flow with different final edge weight cutoff thresholds. In (**a**), with cutoff threshold set between 1 and 0.47 as the range highlighted in blue, we detected all communities correctly. In (**b**), we chose the cutoff threshold to be the turning point of modularity at *w* = 3.2 as the middle vertical line. To compare the communities detected with different cutoff thresholds, two extra cutoff *w* = 3.9 and *w* = 1 are added. The detected communities are shown in Fig. 7.

**Figure 7:**
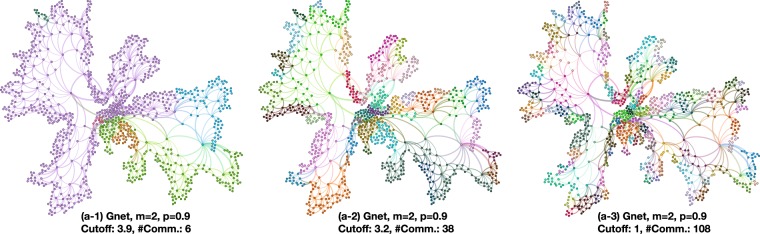
Illustrations of communities detected by discrete Ricci flow with three different cutoff weights of a GNet planar model with 1000 nodes, *m* = 2, and *p* = 0.9. With different cutoff thresholds (labeled as vertical lines in Fig. 6(b)), we are able to detect communities in a hierarchical manner.

To remove the singularities generated during the Ricci flow, we applied the surgery which removed edges with weight greater than an intermediate cutoff threshold for every 5 iterations during the whole 50 iteration process. The clustering accuracy results under different accuracy metrics are shown in Fig. 6. In Fig. 6(a), when the (final) cutoff threshold is set between 1 and 0.47, we have a perfect clustering result of detecting all 30 communities, and this is correctly captured by ARI with the highest possible score 1.0. (In classical case of Hamilton-Perelman Ricci flow on 3-manifolds, the time to do surgery depends on individual manifolds) For modularity, the trend of capturing the perfect clustering accuracy result is similar to ARI (before the cutoff threshold 0.47), but its highest score occurs with a cutoff threshold of 0.275, which detected 290 communities. With this connection that ARI and modularity tend to capture the communities in the same trend, hence for network without community labels such as GNet, a cutoff threshold is suggest to be when modularity first hits the plateau of the curve, for example with cutoff at 3.2 in Fig. 6(b). This cutoff threshold also gives us a hint to detect hierarchical community structures. In Fig. 7, layered community structures are revealed by applying different cutoff thresholds after 20 iterations of discrete Ricci flow processes.

### Comparison with other methods

We compared our result with the community detection algorithms such as Modularity based Fast Greedy algorithm^7^, Label Propagation^12^, Infomap^13^, Spinglass^53^, and Edge Betweenness^14^ (by iGraph: http://igraph.org/python/) with Adjusted Rand Index (ARI) as the accuracy metric.

We first tested community detection algorithms on a simple graph model SBM with 500 nodes, 6800 edges and two even sized communities in Fig. 5(a). We fixed *P*_*inter*_ = 0.15 and tested the mixing ratio *P*_*intra*_/*P*_*inter*_ from 0.1 to 0.9. For SBM, beside label propagation method and Infomap, most of the algorithms perform well when the mixing ratio is below 0.5.

For LFR graphs, Ricci flow and Spinglass outperform all other methods in our experiments (Fig. 5(b)). Compared to the accuracy of 95% for Spinglass, Ricci flow is more stable with nearly perfect accuracy for most of the values of *μ*. We also evaluated community detection algorithms on different real-world datasets. In Fig. 5(c), Ricci flow shows competitive or better results in Karate club, Football, Polbooks, and Polblogs datasets.

**Figure 8:**
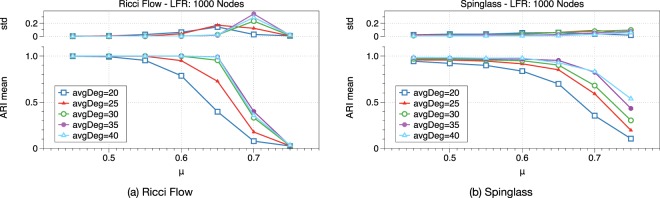
Ricci flow and spinglass algorithms on LFR graphs with different average node degrees. With a higher average degree which implies higher edge density within communities, both algorithms provide better clustering results.

One key factor of a community structure is the density of connections within communities, the community structure is stronger if nodes in one community are more densely connected. In Fig. 8, we tested Ricci flow and spinglass on LFR graphs with different average degree settings. The results show that with a higher average degree (higher edge density within communities) both algorithms provide better clustering results.

## Conclusion

In this paper, we have introduced geometric tools to investigate the community structures on complex networks. The basic idea is to consider networks as geometric objects and use the notion of curvature and curvature guided flow to decompose networks. In classical mathematics, Ricci curvature and Ricci flow are among the most important tools for analyzing and decomposing manifolds according to their geometric and topological properties. What is interesting is that the corresponding discrete counterparts are shown to be powerful for detecting community structures. Interesting future work includes improving the theoretical understanding of discrete curvature on graphs and applying our methods for real-world applications.

## Supplementary information

Supplementary Information

## Data Availability

The datasets generated during and/or analyzed during the current study are available from the corresponding author on reasonable request.

## References

[1] Bhowmick SS, Seah BS (2015). Clustering and summarizing protein-protein interaction networks: A survey. IEEE Trans. Knowl. Data Eng..

[2] Yang Z, Algesheimer R, Tessone CJ (2016). A comparative analysis of community detection algorithms on artificial networks. Sci. Rep..

[3] Fortunato S (2010). Community detection in graphs. Phys. Rep..

[4] Newman MEJ (2006). Modularity and community structure in networks. Proc. Natl. Acad. Sci. USA.

[5] Sinha A, Gleich DF, Ramani K (2018). Gauss’s law for networks directly reveals community boundaries. Sci. Rep..

[6] Leskovec, J., Lang, K. J. & Mahoney, M. Empirical comparison of algorithms for network community detection. In *Proc. 19th Int. Conf. World Wide Web*, 631–640 (ACM, 2010).

[7] Clauset A, Newman MEJ, Moore C (2004). Finding community structure in very large networks. Phys. Rev. E.

[8] Zhang P, Moore C (2014). Scalable detection of statistically significant communities and hierarchies, using message passing for modularity. Proc. Natl. Acad. Sci..

[9] Peel L, Larremore DB, Clauset A (2017). The ground truth about metadata and community detection in networks. Sci. Adv..

[10] Allen B (2017). Evolutionary dynamics on any population structure. Nature.

[11] Abbe E (2018). Community detection and stochastic block models: Recent developments. J. Mach. Learn. Res..

[12] Raghavan UN, Albert R, Kumara S (2007). Near linear time algorithm to detect community structures in large-scale networks. Phys. Rev. E Stat. Nonlin. Soft Matter Phys..

[13] Rosvall M, Bergstrom CT (2008). Maps of random walks on complex networks reveal community structure. Proc. Natl. Acad. Sci. USA.

[14] Girvan M, Newman MEJ (2002). Community structure in social and biological networks. Proc. Natl. Acad. Sci. USA.

[15] Newman ME, Girvan M (2004). Finding and evaluating community structure in networks. Phys. Rev. E.

[16] Hamilton RS (1982). Three-manifolds with positive ricci curvature. J. Differ. Geom..

[17] Perelman, G. The entropy formula for the ricci flow and its geometric applications, https://arxiv.org/abs/math/0211159 (2002).

[18] Jost, J. *Riemannian geometry and geometric analysis* (Springer Science & Business Media, 2011).

[19] Ollivier Y (2009). Ricci curvature of markov chains on metric spaces. J. Funct. Anal..

[20] Ollivier, Y. A survey of ricci curvature for metric spaces and markov chains. In *Probabilistic Approach to Geometry*, 343–381, 10.2969/aspm/05710343 (Math. Soc. of Japan, Tokyo, Japan, 2010).

[21] Lott J, Villani C (2009). Ricci curvature for metric-measure spaces via optimal transport. Annals Math. Second. Ser..

[22] Ni, C.-C., Lin, Y.-Y., Gao, J., Gu, X. D. & Saucan, E. Ricci curvature of the internet topology. In *IEEE. Ic. Comp. Com. Net. (INFOCOM)*, vol. 26, 2758–2766, 10.1109/INFOCOM.2015.7218668 (IEEE, 2015).

[23] Samal A (2018). Comparative analysis of two discretizations of Ricci curvature for complex networks. Sci. Rep..

[24] Sreejith RP, Mohanraj K, Jost J, Saucan E, Samal A (2016). Forman curvature for complex networks. J. Stat. Mech: Theory Exp..

[25] Wang, C., Jonckheere, E. & Banirazi, R. Wireless network capacity versus Ollivier-Ricci curvature under Heat-Diffusion (HD) protocol. In *2014 American Control Conference*, 3536–3541 (IEEE, 2014).

[26] Whidden C, Matsen FA (2017). Ricci–Ollivier curvature of the rooted phylogenetic subtree–prune–regraft graph. Theor. Comput. Sci..

[27] Jost J, Liu S (2014). Ollivier’s Ricci curvature, local clustering and Curvature-Dimension inequalities on graphs. Discret. Comput. Geom..

[28] Sandhu R (2015). Graph curvature for differentiating cancer networks. Sci. Rep..

[29] Sandhu RS, Georgiou TT, Tannenbaum AR (2016). Ricci curvature: An economic indicator for market fragility and systemic risk. Sci Adv.

[30] Ni, C. -C., Lin, Y. -Y., Gao, J. & Gu, X. Network alignment by discrete Ollivier-Ricci flow. In *Graph Drawing and Network Visualization*, 447–462 (Springer International Publishing, 2018).

[31] Lancichinetti A, Fortunato S, Radicchi F (2008). Benchmark graphs for testing community detection algorithms. Phys. Rev. E.

[32] Bianconi G, Darst RK, Iacovacci J, Fortunato S (2014). Triadic closure as a basic generating mechanism of communities in complex networks. Phys. Rev. E Stat. Nonlin. Soft Matter Phys..

[33] Wu Z, Menichetti G, Rahmede C, Bianconi G (2015). Emergent complex network geometry. Sci. reports.

[34] Hubert L, Arabie P (1985). Comparing partitions. J. Classif..

[35] Saucan E, Samal A, Weber M, Jost J (2018). Discrete curvatures and network analysis. MATCH Commun. Math. Comput. Chem..

[36] Sreejith RP, Jost J, Saucan E, Samal A (2017). Systematic evaluation of a new combinatorial curvature for complex networks. Chaos Solitons Fractals.

[37] Bakry, D. & Émery, M. Diffusions hypercontractives. In Azéma, J. & Yor, M. (eds) *Séminaire de Probabilités XIX 1983/84*, vol. 1123 of *Lecture Notes in Mathematics*, 177–206 (Springer Berlin Heidelberg, Berlin, Heidelberg, 1985).

[38] Bonciocat, A. I. & Sturm, K. T. Mass transportation and rough curvature bounds for discrete spaces. *J. Funct. Anal* (2009).

[39] Bonciocat A-I (2014). A rough curvature-dimension condition for metric measure spaces. Cent. Eur. J. Math..

[40] Wang, C., Jonckheere, E. & Banirazi, R. Interference constrained network control based on curvature. In *Proc. American Control Conference*, vol. 2016-July, 6036–6041 (IEEE, 2016).

[41] Pal, S. *et al*. Jaccard curvature—an efficient proxy for Ollivier-Ricci curvature in graphs. In *Complex Networks IX*, 51–63 (Springer International Publishing, 2018).

[42] Forman R (2003). Bochner’s method for cell complexes and combinatorial ricci curvature. Discret. Comput. Geom..

[43] Weber M, Saucan E, Jost J (2017). Characterizing complex networks with Forman-Ricci curvature and associated geometric flows. J Complex Netw.

[44] Weber, M., Jost, J. & Saucan, E. Detecting the coarse geometry of networks. In *NeurIPS 2018 Workshop*, https://www.mis.mpg.de/preprints/2018/preprint2018_97.pdf (2018).

[45] Saucan, E., Wolansky, G., Appleboim, E. & Zeevi, Y. Y. Combinatorial ricci curvature and laplacians for image processing. In *2nd Int. Cong. on Image and Signal Processing*, 1–6, 10.1109/CISP.2009.5304710 (2009).

[46] Chow B (2003). Combinatorial Ricci flows on surfaces. J. Differ. Geom..

[47] Plantié, M. & Crampes, M. Survey on social community detection. In *Social Media Retrieval*, Computer Communications and Networks, 65–85 (Springer, London, 2013).

[48] Parés, F. *et al*. Fluid communities: A competitive, scalable and diverse community detection algorithm. In *Complex Networks & Their Applications VI*, 229–240 (Springer International Publishing, 2018).

[49] Yin H, Benson AR, Leskovec J, Gleich DF (2017). Local higher-order graph clustering. ACM Trans. on Knowl. Discov. from Data (TKDD).

[50] Newman MEJ (2016). Equivalence between modularity optimization and maximum likelihood methods for community detection. Phys. Rev. E.

[51] Decelle A, Krzakala F, Moore C, Zdeborová L (2011). Asymptotic analysis of the stochastic block model for modular networks and its algorithmic applications. Phys. Rev. E.

[52] Ji J, Zhang A, Liu C, Quan X, Liu Z (2014). Survey: Functional module detection from protein-protein interaction networks. IEEE Trans. Knowl. Data Eng..

[53] Reichardt J, Bornholdt S (2006). Statistical mechanics of community detection. Phys. Rev. E.

[54] Faqeeh A, Osat S, Radicchi F (2018). Characterizing the analogy between hyperbolic embedding and community structure of complex networks. Phys. Rev. Lett..

[55] Salnikov V, Cassese D, Lambiotte R (2018). Simplicial complexes and complex systems. Eur. J. Phys..

[56] Lin Y, Lu L, Yau S-T (2011). Ricci curvature of graphs. Tohoku Math. J..

[57] Kunegis, J. KONECT: The koblenz network collection. In *Proceedings of the 22Nd International Conference on World Wide Web*, WWW ’13 Companion, 1343–1350 (ACM, New York, NY, USA, 2013).

[58] Leskovec, J. & Krevl, A. SNAP Datasets: Stanford large network dataset collection, http://snap.stanford.edu/data (2014).

[59] Bastian, M., Heymann, S. & Jacomy, M. Gephi: An open source software for exploring and manipulating networks. *Int. AAAI Conf. on Weblogs Soc. Media* (2009).

