# Author Correction: Community Detection on Networks with Ricci Flow

**DOI:** 10.1038/s41598-019-49491-5

**Published:** 2019-09-03

**Authors:** Chien-Chun Ni, Yu-Yao Lin, Feng Luo, Jie Gao

**Affiliations:** 1 Yahoo! Research, Sunnyvale, CA USA; 2 Intel Inc., Hillsboro, OR USA; 3 Rugters University, New Brunswick, NJ USA; 4 Stony Brook University, Stony Brook, NY USA

Correction to: *Scientific Reports* 10.1038/s41598-019-46380-9, published online 10 July 2019

This Article contains an error in the Data Availability section, where the link to the code repository is missing. As a result,

“The datasets generated during and/or analyzed during the current study are available from the corresponding author on reasonable request.”

should read:

“The datasets generated during and/or analyzed during the current study are available from the corresponding author on reasonable request. Code for graph Ricci curvature and Ricci flow computation is available on GitHub (https://github.com/saibalmars/GraphRicciCurvature).”

