# Supplementary material for: Community Detection on Networks with Ricci Flow
Source: Sci Rep. 2019 Jul 10;9:9984. doi: 10.1038/s41598-019-46380-9 (PMC6620345; doi:10.1038/s41598-019-46380-9)
Supplement: Supplementary file 1 — Supplementary Information [file 41598_2019_46380_MOESM1_ESM.pdf]

SUPPLEMENTARY INFORMATION
Community Detection on Network with Ricci Flow

Chien-Chun Ni[1], Yu-Yao Lin[2], Feng Luo[3], and Jie Gao[4,*]

[1]Yahoo! Research, Sunnyvale, CA, USA
[2]Intel Inc., Hillsboro, OR, USA
[3]Rugters University, New Brunswick, NJ, USA
[4]Stony Brook University, Stony Brook, NY, USA
[*]jgao@cs.stonybrook.edu

1 Theoretical foundation

We model a network as an unweighted graph $G = (V, E)$ with node set V and edge set E. The community detection problem on G is to find a collection of edges, called *inter-communities edges*, such that removing these edges from G produces a collection of connected subgraphs $C_1, ..., C_n$, called *communities*, with the following properties (1) nodes in each community C_i are heavily connected by *intra-community edges* among themselves and (2) nodes in different communities are sparsely connected. It has been observed in many real-world networks (e.g., social, biological, the Internet) that communities exist. Detecting communities in a network has become an important and fundamental problem in complex networks with many applications. However, from a mathematical point of view, a precise and universally accepted definition of community structure is still lacking.

Our approach to define communities in a network uses the notion of Ricci curvature in geometry and the mathematical theory of optimal transport. Consider a graph G as a transportation network, edges between different communities are heavily traveled compared to edges within a community, since traffic prefers to take shortest paths whenever possible. Specifically, one can quantify the amount of traffic through an edge using the transportation cost from the optimal transportation theory.

We will use the following definitions and conventions. Two vertices (or nodes) $i, j \in V$ are adjacent, denoted by $i \sim j$, if they are the endpoints of an edge. In this case, we denote the edge by ij. The edge weight $w : E \to \mathbb{R}_{\geq 0}$ on G assigns each edge ij a non-negative number w_{ij}. The triple (V, E, w) is called a weighted graph or a metric graph. A path from node a to node b is a collection of edges $e_i = v_i v_{i+1}$ for $i = 0, 1, 2, ..., n-1$ such that $v_0 = a$ and $v_n = b$. The *length* of the path $\{e_0, e_1, ..., e_{n-1}\}$ is defined to be $\sum_{i=0}^{n-1} w_{i(i+1)}$. The path is said to have n hops.

1.1 The optimal transport problem

The original optimal transport problem considered by G. Monge in 1781 is to minimize the transportation cost to move iron ores in different mines to a collection of factories which consume the iron ores. Mathematically, the problem is formulated as follows. To begin, let us briefly recall the notion of metric spaces and Borel measures on a metric space. A metric space is a pair (X, d) where X is a set and d is a distance function $d : X \times X \to \mathbf{R}_{\geq 0}$ with the following properties:

- $d(x, y) = 0$ if and only if $x = y$;
- $d(x, y) = d(y, x)$;
- $d(x, y) + d(y, z) \geq d(x, z)$ for all $x, y, z \in X$.

Given a metric space (X, d), a Borel set in X is obtained from open sets in X through the operations of countable union, countable intersection, and relative complement. A Borel probability measure μ on a metric space (X, d) assigns each Borel set A a non-negative real number $\mu(A)$ such that

- $\mu(X) = 1$;
- if $A_, ..., A_n, ...$ are disjoint Borel sets then $\mu(\cup_{i=1}^{\infty} A_i) = \sum_{i=1}^{\infty} \mu(A_i)$.

In our case of a finite graph $G = (V, E)$, we take X to be the vertex set V. Every subset of X is Borel. A Borel probability measure can be identified with a function $\mu : V \to [0,1]$ such that $\sum_{i \in V} \mu(i) = 1$. Given an edge weighted graph (V, E, w) with $w_{ij} > 0$ for all edges, one introduces a metric d on vertex set V by

$$d(v, v') = \min\{\sum_{i=0}^{n} w_{k_i,k_{i+1}} : k_i \in V, k_0 = v, k_{n+1} = v', k_i \sim k_{i+1}\} \tag{1}$$

where the minimum is taken over all edge paths from v to v'. We call d the induced metric from the edge weight w. Note that by definition, $d(v, v') + d(v', v) \geq d(v, v'')$ for any three vertices $v, v', v'' \in V$.

Let X and Y be two metric spaces and μ and ν be two probability Borel measures on X and Y respectively. Here X and Y stand for mines and factories and μ and ν are the amount of iron ores to be moved and consumed respectively. Let $c : X \times Y \to \mathbb{R}_{\geq 0}$ be a continuous function considered as the cost, i.e., the cost of transporting from location x to location y is $c(x, y)$. The function c is usually taken to be the distance $d(x, y)$ if $X = Y$ and the cost of transportation per-unit distance is constant.

A *transport map* $T : (X, \mu) \to (Y, \nu)$ is a measure preserving map, i.e., for any Borel set $A \subset Y$, $\nu(A) = \mu(T^{-1}(A))$. Monge's formulation of the optimal transportation problem is to find a transport map $T : X \to Y$ that realizes the infimum

$$\inf\left\{ \int_X c(x, T(x))\, \mathrm{d}\mu(x) \,\middle|\, \text{T is a transportation} \right\}.$$

A map T that attains this infimum (i.e. makes it a minimum instead of an infimum) is called an "optimal transport map". In this generality, there is no mathematical theorem that guarantees the existence of the optimal transportation map. A breakthrough in optimal transportation problem was made by L. Kantorovich in 1942 [1]. In this paper, he formulated the optimal transportation problem as a linear optimization problem whose solution always exists. He replaced the transportation map T by the *transportation plans* which are probability measures γ on the product space $X \times Y$ satisfying $\gamma(A \times Y) = \mu(A)$ and $\gamma(X \times B) = \nu(B)$ for all Borel sets A and B. The goal is to find a transportation plan γ that attains the infimum cost

$$W(\mu, \nu) = \inf\left\{ \int_{X \times Y} c(x, y)\, \mathrm{d}\gamma(x, y) \,\middle|\, \gamma \in \Gamma(\mu, \nu) \right\},$$

where $\Gamma(\nu, \mu)$ denotes the collection of all transportation plans. If $X = Y$, the quantity $W(\mu, \nu)$ is called the *Wasserstein distance* between two probability measures μ, ν on X. Kantorovich proved that the infimum is always achieved by some transportation plan.

In our case of a finite weighted graph $G = (V, E, w)$ with two probability measures μ and ν on the vertex set V, one can reformulate Kantorovich's problem as follows. Let d be the associated distance function on V defined by Equation (1). A transportation plan γ is given by a map $\gamma : V \times V \to [0,1]$ such that $\sum_{i \in V} \gamma_{ij} = \mu_j$ and $\sum_{j \in V} \gamma_{ij} = \nu_i$ for all $i, j \in V$. The goal is to find the minimum cost

$$\min\{ \sum_{i,j \in V} \gamma_{ij} d(i, j) : \ \gamma \text{ is a transportation plan} \}.$$

This is a linear programming problem and thus can be computed.

1.2 Curvatures in classical differential geometry

One of the central themes in modern geometry is the notion of curvature which measures how space is curved. It was introduced by Gauss and Riemann over 190 years ago. By an n-dimensional manifold, following Riemann, we mean a space which locally looks like the n-dimensional space. A Riemannian metric on a manifold assigns each tangent space of the manifold a Euclidean metric. Manifolds together with Riemannian metrics, i.e., Riemannian manifolds, are the main objects of study in modern geometry. For instance, a smooth surface in the 3-dimensional Euclidean space is a 2-dimensional Riemannian manifold whose associated Riemannian metric is induced from the 3-dimensional Euclidean space. These are the original objects investigated by Gauss. For such a surface S, the *Gaussian map* from S to the unit sphere sends a point on S to the unit normal vector of S at p. The *curvature* (or Gaussian curvature) of the surface at a point p is the signed area distortion of the Gaussian map at p. To be more precise, it is the Jacobian determinant of the Gaussian map at p. From this definition, one sees that the plane has zero curvature, the sphere has positive curvature, and the hyperboloid of one sheet has negative curvature. Gauss showed (Theorema egregium) that

the curvature depends only on the induced Riemannian metric on the surface and does not depend on how the surface is embedded. Riemann generalized Gaussian curvature to high dimensions as follows. For a Riemannian manifold (M,g), the *sectional curvature* assigns each 2-dimensional linear subspace P in the tangent space T_pM of M at p a scalar (the Riemannian sectional curvature). The scalar is equal to the Gaussian curvature of the image of P under the exponential map. A positively curved space tends to have small diameter and is geometrically crowded (e.g., a sphere). In contrast, a negatively curved space has an infinite fundamental group, a contractible universal cover, and is geometrically spreading out like a tree (in large scale).

The *Ricci curvature* assigns each unit tangent vector v at a point p a scalar which is the average of the sectional curvatures of planes containing v. There are several characteristic properties of the Ricci curvature which are used for defining the Ricci curvature on general metric spaces. Namely, Ricci curvature controls how fast the volume of distance ball grows as a function of the radius. It also controls the volume of the overlap of two balls in terms of their radii and the distance between their centers.

There have been various approaches to generalize the notion of curvature to spaces which are not manifolds (e.g., a graph with edge weights). One of the important work of Ollivier [2] Ricci curvature relates to optimal transport. Since optimal transport can be formulated on very general metric spaces with probability measure at each point, in particular on networks with edge weights and probability measures at each vertex. This is the approach we take for community detection using curvature and optimal transport.

1.3 Ollivier's work on Ricci curvature for general metric spaces with measures

Ollivier's approach to Ricci curvature relies on the following key theorem [2] which relates curvature with optimal transportation. Let (M^n,d) be an n-dimensional Riemannian manifold with Riemannian distance d whose Ricci curvature is k and Riemannian volume measure is μ. Fix $\varepsilon > 0$, let

$$m_x = \frac{\mu|_{B(x,\varepsilon)}}{\mu(B(x,\varepsilon))} \tag{2}$$

be the probability measure associated to $x \in M$. Then the Wasserstein distance $W(m_x,m_y)$ is

$$(1-k(x,y))d(x,y),\; k_{xy} = \frac{\varepsilon^2 k(v,v)}{2(n+2)} + O(\varepsilon^3 + \varepsilon^2 d(x,y))$$

and v is the tangent vector at x of the geodesic xy. This shows that the classical Ricci curvature is related to the optimal transportation problem. This also shows how to define Ricci curvature for general metric spaces with probability measures.

Definition 1.1. *(Ollivier [2]) Given a metric space (X,d) equipped with a probability measure m_x for each $x \in X$, the Ollivier's Ricci curvature along the shortest path xy is*

$$k(x,y) = 1 - \frac{W(m_x,m_y)}{d(x,y)} \tag{3}$$

where $W(m_x,m_y)$ is the Wasserstein distance with respect to the cost function $c(x,y) = d(x,y)$.

1.4 Ricci curvature for weighted graph

In our case of weighted graph, we adapt Ollivier's definition of discrete Ricci curvature and relate it to community detection. On a weighted graph (V,E,w) we consider the associated distance function d defined by Equation (1). To define the Ollivier Ricci curvature, one needs probability measures associated with each vertex. In [3], given a non-negative scalar α, Lin *et al.* defined the probability measure m_x^α on V associated to the vertex $x \in V$ to be

$$m_x^\alpha(i) = \begin{cases} \alpha & \text{if } i = x \\ (1-\alpha)/|\pi(x)| & \text{if } i \sim x \\ 0 & \text{otherwise,} \end{cases} \tag{4}$$

where $\pi(x)$ is the set of neighbors of x and $|\pi(x)|$ is the number of elements in set. In this work, we extended and generalized the probability distribution defined by Lin *et al.* to further consider the edge weights. The probability measure we constructed is motivated by the classical differential geometry as follows. Consider a network as a

discretization of a smooth manifold, a Riemannian metric and a Riemannian distance should correspond to edge weight and distance. Given an edge weighted graph (V,E,w) whose associated distance is d (Equation (1)), we define the associated normalized probability measure (Equation (2)) as follows.

$$m_x^{\alpha,p}(i) = \begin{cases} \alpha & \text{if } i = x \\ \frac{1-\alpha}{C} \cdot \exp(-(d(x,i))^p) & \text{if } i \sim x \\ 0 & \text{otherwise,} \end{cases} \tag{5}$$

where C is a normalization factor $C = \sum_{j \sim x} \exp(-(d(x,j))^p)$. Then the discrete Ricci curvature defined under this mass distribution is as follows:

$$\kappa_{xy} = 1 - \frac{W(m_x^{\alpha,p}, m_y^{\alpha,p})}{d(x,y)} \tag{6}$$

Note that for $p = 0$, the probability measure becomes $m_x^{\alpha,0}(i) = \frac{1-\alpha}{|\pi(x)|}$ if $i \sim x$, which is the same as Equation (4). For our computation, we take $p = 2$ and $\alpha = 1/2$ in most cases.

To detect the community structure, we introduce a curvature guided diffusion process called discrete Ricci flow (or simply Ricci flow) on a network. The flow was motivated by the powerful tool of smooth Ricci flow which has recently revolutionized the geometry and topology of 3-dimensional spaces. It is also related to the work of discrete Ricci flow on surfaces [4].

The Ricci flow on weighted graph $(V,E,w^{(0)})$ generates a time dependent family of weighted graphs $(V,E,w(t))$ such that the initial value $w(0) = w^{(0)}$ and the weight $w_{ij}(t)$ on edge ij changes proportional to the Ollivier-Ricci curvature $\kappa_{ij}(t)$ at edge ij at time t.

Ollivier, in the future work section of [2], suggested to use the following formula for Ricci flow with continuous time parameter t:

$$\frac{d}{dt} d_{ij}(t) = -\kappa_{ij}(t) d_{ij}(t). \tag{7}$$

In our setting, we use discrete time parameter k and define a discrete Ricci flow as a family of edge weighted graphs $(V,E,w^{(k)})$, $k \in \mathbf{Z}_{\geq 0}$, with associated distances $d^{(k)}$, where $d^{(k)}(i,j)$ is the shortest edge path length between i and j. The flow takes discrete time $k \in \mathbf{Z}_{\geq 0}$.

$$w_{ij}^{(k+1)} = (1 - \kappa_{ij}^{(k)}) d^{(k)}(i,j) \tag{8}$$

where $\kappa_{ij}^{(k)}$ is the Ricci curvature at the edge ij of the weighted graph $(V,E,w^{(k)})$. Note that Equation (8) states that $w_{ij}^{(k+1)}$ is equal to the Wasserstein distance between adjacent vertices i and j in the metric graph $(V,E,w^{(k)})$. To detect a community structure on an unweighted graph $G = (V,E)$, we take the initial edge weight $w^{0)}$ to be the constant 1 and run the Ricci flow (Equation (8)) from there.

The flow tends to expand negatively Ricci curved subgraphs and contract positively Ricci curved subgraphs. In networks with clear community structures, the inter-community edge weights converge to ∞ and the intra-community edge weights converge to 0. Thus the network is naturally partitioned into different communities by removing edges of very high weights. We may continue to run Ricci flow to further partition the communities into smaller ones, when the network has hierarchical community structures. This is similar to the popular Girvan-Newman algorithm [5]. It first computes betweenness centrality for each edge, then removes edges with the highest score. After that, re-compute all scores and repeat. Here the betweenness of an edge is the sum $\sum_{i,j \in V\{\partial e\}} \frac{\sigma_{ij}(e)}{\sigma_{ij}}$ where σ_{ij} is the number of shortest paths from i to j and $\sigma_{ij}(e)$ is the number of shortest paths from i to j which contain the edge e. Since computing centrality involves global information of the network and is expensive, Ricci flow is easier to implement in practice.

Our work on Ricci curvature on networks builds on our previous work [6] and is also inspired by the important works of E. Saucan and J. Jost *et al.* in [7, 8, 9, 10]. In these works, they systematically introduced and investigated various discrete curvatures for complex networks. The comparative analysis of Forman and Ollivier Ricci curvature on benchmark dataset of complex networks and real-world networks was also carried out. Their numerical results show a striking fact that these two completely different discretization of the Ricci curvatures are highly correlated in many networks.

2 From Ricci flow to community detection

In this section, we explain the relationship between the discrete Ricci flow (Equation 8) on networks and the classical theory of 3-manifolds and the Ricci flow. These classical theories motivate us to apply Ricci flow for community detection.

A 3-manifold in mathematics is a connected (Hausdorff) topological space which looks like the 3-space in small scale. More precisely, each point in a 3-manifold has a neighbourhood homeomorphic to an open ball in the 3-space. 3-manifolds are the basic prototypes of 3-dimensional spaces. There are two operations which produce complicated 3-manifolds from simple ones. The first is the connected sum operation and the second is the torus sum. In the connected sum operation, one takes two 3-manifolds, removes two small open balls from them. Glue the two remaining 3-manifolds with holes along their 2-sphere boundary by a homeomorphism. The resulting 3-manifold is the connected sum of the given two. The torus sum operation is similar where one takes two 3-manifolds with torus boundary and glues the two boundary by a homeomorphism. Naturally, one asks if each 3-manifold can be decomposed as a connected sum and torus sum from simple pieces, i.e., reversing the above process. The answer is affirmative and is the content of the classical theorem of topological decomposition of 3-manifolds, established by H. Kneser, J. Milnor [11] Jaco-Shalen [12], and Johannson [13]. The theorem states that each 3-manifold can be canonically decomposed into simple pieces using the 2-spheres and tori. In the sphere decomposition, one looks for topologically essential disjoint 2-spheres in the 3-manifold. Here a topologically essential 2-sphere (or torus) means it is not the boundary of a 3-ball (or a solid torus) in the manifold. By capping off the 2-sphere boundary of the compliment by the 3-balls, one obtains new 3-manifolds which have simpler topology and the original 3-manifold is obtained from them by the connected sum. The torus decomposition is similar in construction and uses the essential torus instead of the 2-sphere. A 3-manifold which cannot be decomposed by any essential 2-spheres and tori is called atoroidal. Thus the classical decomposition theorem says that each 3-manifold is a connected sum and torus sum of a collection of atoroidal ones.

One of the most important problems in low-dimensional geometry and topology is the geometrization conjecture proposed by William Thurston in 1976 [14]. It states that any atoroidal 3-manifold admits complete, locally homogeneous Riemannian metrics, i.e., atoroidal 3-manifolds are geometric. This fundamental conjecture was solved by the groundbreaking work of Perelman in 2004 which uses the Ricci flow method developed by R. Hamilton in 1981. A key step in Perelman's proof is that the Ricci flow can detect the 2-sphere and torus decomposition.

The Ricci flow, introduced by Richard S. Hamilton in 1981 [15], deforms the metric of a Riemannian manifold in a way formally analogous to the diffusion of heat, smoothing out irregularities in the metric. Ricci flow has been one of the most powerful tools for solving geometric problems in the past forty years. The flow exhibits many similarities with the heat equation.

Suppose a Riemannian metric g_{ij} is given on a manifold M so that its Ricci curvature is R_{ij}. Then Hamilton's Ricci flow is the following second-order nonlinear partial differential equation on symmetric $(0,2)$-tensors:

$$\frac{\partial}{\partial t} g_{ij} = -2R_{ij}.$$

A solution to the Ricci flow is a one-parameter family of metrics $g_{ij}(t)$ on a smooth manifold M satisfying the above partial differential equation. One of the key properties of the Ricci flow is that the curvature evolves according to a nonlinear version of heat equation. Thus the Ricci flow tends to smoothing out irregularity of the curvature.

In the groundbreaking work of G. Perelman, he used the Ricci flow to prove the geometrization conjecture by analyzing the singularity formation and introducing a Ricci flow with 'surgery' [16]. One way a singularity may arise in the Ricci flow is that an essential 2-sphere in the manifold may collapse to a point in finite time. Also essential tori in the manifold can also be detected by the Ricci flow. Thus, one consequence of Perelman's work is that Ricci flow can be used to find the 2-sphere and torus decomposition in a 3-manifold. This is the key motivation for us to introduce a discrete Ricci flow on networks for community detection.

We consider a community structure in a network heuristically to be a discrete counterpart of the (topological) sphere-torus decomposition of a 3-manifold. One can roughly justify this analogy as follows. A commonly agreed characterization of communities in a network is that there are more edges linking nodes within a community and fewer edges linking nodes in different community. This is similar to the components in the sphere decomposition. Indeed, according to the Seifert-van Kampen theorem ([17]), loops within a component in the sphere and torus decomposition interact more among themselves than loops in different components. Since the Ricci flow runs on a manifold with a Riemannian metric and detects topological decompositions, the discrete Ricci flow on a network with edge weight should detect the community structures. This theme has been supported by our numerical experiments.

3 Algorithms for discrete Ricci flow on graph

For a given graph $G = (V, E)$, let the edge weight of edge $xy \in E$ be w_{xy}, the discrete Ricci curvature of edge xy, κ_{xy}, is computed as follows:

$$\kappa_{xy} = 1 - \frac{W(m_x^{\alpha,p}, m_y^{\alpha,p})}{d(x,y)},$$

where W is the optimal mass transport distance (a.k.a. Wasserstein Distance or Earth Mover Distance). The mass distribution $m^{\alpha,p}$ is defined as

$$m_x^{\alpha,p}(i) = \begin{cases} \alpha & \text{if } i = x \\ \frac{1-\alpha}{C} \cdot \exp(-(d(x,i))^p) & \text{if } i \sim x \\ 0 & \text{otherwise,} \end{cases}$$

where C is a normalization factor $C = \sum_{j\sim x} \exp(-(d(x,j))^p)$. To speed up the computation of W, we suggest to apply the Sinkhorn distance [18] which computes the approximate optimal transport distance fast without losing too much accuracy.

For discrete Ricci flow computation, we follow Equation 8. For each iteration i, the Ricci flow metric on edge (edge weight) is defined as follows:

$$w_{xy}^{(i+1)} = d^{(i)}(x,y) - \varepsilon \cdot \kappa_{xy}^{(i)} \cdot d^{(i)}(x,y), \quad \forall xy \in E,$$

where ε is the step size or learning rate of the gradient decent process. $d^{(i+1)}(x,y)$ is the shortest path between x and y based on $w^{(i+1)}$.

Since the edge weights change in a relative manner, we re-scale the metric so that the sum of edge weight remains constant. For each iteration, we recompute the Ricci curvature on each edge based on the current edge weight, then update and normalize the weight until $|\kappa_{xy}^{(i)} - \kappa_{xy}^{(i-1)}| < \delta$, where $\delta > 0$. The detailed algorithm is as follows.

Algorithm 1: Discrete Ricci Flow

Input : An undirected graph G and a real number $\delta > 0$.
Output : A weighted graph G with edge weight as Ricci flow metric on each edge.

1. Normalize the edge weight $w_{xy}^{(i)} \leftarrow d^{(i)}(x,y) \cdot \frac{|E|}{\sum_{xy\in E} d^{(i)}(x,y)}$
2. Compute the Ricci curvature of G
3. Update the edge weight by $w_{xy}^{(i+1)} \leftarrow d^{(i)}(x,y) - \varepsilon \cdot \kappa_{xy}^{(i)} \cdot d^{(i)}(x,y)$
4. Repeat $1-3$ until all $|\kappa_{xy}^{(i)} - \kappa_{xy}^{(i-1)}| < \delta$.

4 Evaluations

Here we briefly introduce these two metrics and apply these metrics to measure the accuracy of our clustering results.

ARI Adjusted Rand Index (ARI) [19] measures the similarity between two clustering results by adjusting the Rand index [20] in a way that a random result gets a score of 0. Suppose the ground truth community is described by a partition of nodes $\{1, 2, \cdots, n\}$ into disjoint sets $C_1, C_2, \cdots, C_m$, and the detected community is represented by disjoint sets $\{\bar{C}_1, \bar{C}_2, \cdots, \bar{C}_k\}$. ARI scores the agreement of partitioned node pairs in C_i and $\bar{C}_j$. The ARI of the given clustering result and the actual result is defined as follows.

$$ARI(C, \bar{C}) = \frac{\sum_{i=1}^{m}\sum_{j=1}^{k}\binom{|C_i\cap\bar{C}_j|}{2} - [\sum_{i=1}^{m}\binom{|C_i|}{2}\sum_{j=1}^{k}\binom{|\bar{C}_j|}{2}]/\binom{|V|}{2}}{\frac{1}{2}[\sum_{i=1}^{m}\binom{|C_i|}{2} + \sum_{j=1}^{k}\binom{|\bar{C}_j|}{2}] - [\sum_{i=1}^{m}\binom{|C_i|}{2}\sum_{j=1}^{k}\binom{|\bar{C}_j|}{2}]/\binom{|V|}{2}} \quad (9)$$

The value of ARI is 1 when the two clustering results match perfectly and is below 1 otherwise.

Modularity Modularity introduced in [21] describes the fraction of edges between communities minus the expected fraction if edges are distributed uniformly at random. The range of modularity is $[-1,1)$ where a positive value means more edges are intra-community edges than expected for a random graph. Unlike ARI, Modularity does not require ground truth community, and is easy to compute even for large networks. But modularity suffers from resolution problem due to the statistical nature [22].

For a network G with n node, m edges, c communities and adjacency matrix A, the modularity Q of this network is defined as follows:

$$Q = \frac{1}{(2m)} \sum_{vw} \left[A_{vw} - \frac{k_v k_w}{(2m)} \right] \delta(c_v, c_w) = \sum_{i=1}^{c} (e_{ii} - a_i^2),$$

where k_x is the degree of node x, $\delta(c_v, c_w)$ equals to 1 if node v and w are in the same community, otherwise 0, and e_{ij} is the fraction of inter-community edges connecting community i and j:

$$e_{ij} = \sum_{vw} \frac{A_{vw}}{2m} 1_{v \in c_i} 1_{w \in c_j},$$

and a_i is the fraction of intra-community edges in community i:

$$a_i = \frac{k_i}{2m} = \sum_j e_{ij}.$$

4.1 Edge Weights under Ricci Flow Iterations

We first analyze the iterating process of Ricci flow on graphs with community structure. In Fig. 1, we showed the variation of edge weights and edge Ricci curvatures on an LFR graph with 1000 nodes and $\mu = 0.4$ with 38 communities and with average degree 20. We name edges to connect nodes in the same community as intra-community edges (labeled as blue) and edges connect nodes in two different communities as inter-community edges (labeled red in the figure). When the iteration process starts, the original weight of all edges is set to be 1 (Fig. 1(a)). As Ricci flow iterates, we can see a clear trend that the weights of intra-community edges (labeled blue in figure) are decreasing and converged to 0 while the weights inter-community edges increased with iterations. In Fig. 1(b), as Ricci flow iterates, the Ricci curvature on all edges converged to a fix value.

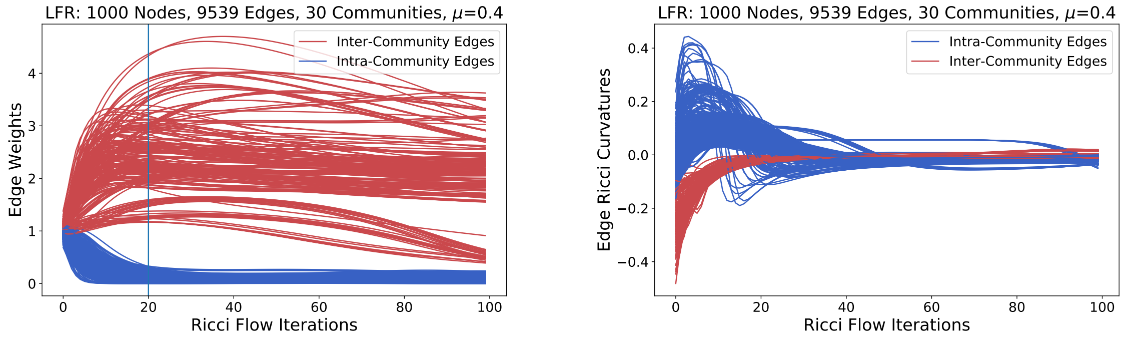

(a) Ricci flow iterations over edge weights

(b) Ricci flow iterations over edge Ricci curvature

Figure 1. Edge weights and Ricci curvatures over Ricci flow iterations on a LFR graph with 1000 nodes, 9539 edges, and $\mu = 0.4$. Fig. 1(a): the edge weights of inter-community edges expand by Ricci flow. Fig. 1(b): edge Ricci curvatures converged to 0 by Ricci flow.

4.2 Ricci Flow Parameters

Here we discuss the influence of different probability measures on Ricci flow. Recall that, when computing the Ricci curvature of each edge, we choose the probability distribution as exponential in the edge weight. This exponential distribution takes *base* to be e and the exponent to be the edge weight to the *power* of p. In Fig. 2, we test the community detection accuracy of Ricci flow using probability distribution with different base and power on LFR graph with 1000 nodes with $\mu = 0.5$ and average degree 20. The result shows that over iterations, choosing $p = 2$ or $p = 3$ yields decent

accuracy. Notice that for $p = 0$, the probability distribution is reduced to Lin and Yau's setting [3] that the mass is equally distributed to a node's neighbors during the optimal transportation process.

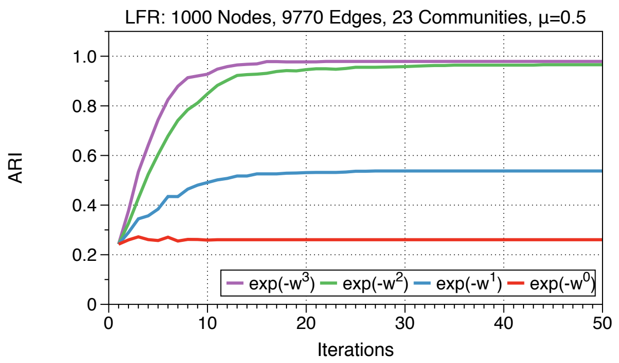

Figure 2. Ricci flow with different power probability measure setting on a LFR graph with 1000 nodes, 9770 edges and $\mu = 0.5$.

In Fig. 3, we check the influence of probability distribution of different *base* with $p = 2$ on LFR graph with 1000 nodes and average degree 20. To eliminate the influence of randomness of the model, for every different μ, we take the average result over 10 trials. The result shows that for most of the *base* setting, the result is good when μ is smaller than 0.6. For a more mixed graph with greater μ, choose *base* to be e yields the most stable result.

In the following experiments, we stick to $base = e$ and $power = 2$ as our parameter setting.

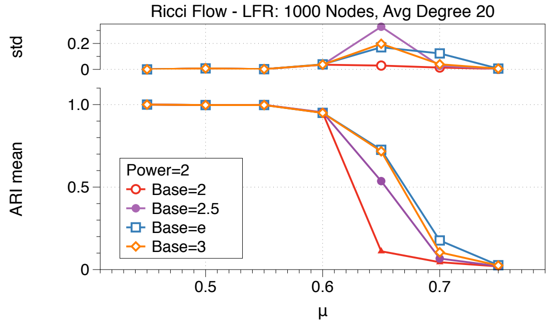

Figure 3. Ricci flow with different base probability measure setting on a LFR graph with 1000 nodes, 9770 edges and $\mu = 0.5$.

4.3 Approximated Optimal Transport Distance

To speed up the computation process, we suggest utilizing the Sinkhorn distance [18] as an approximate solution for optimal transport. The result of applying these two distances for Ricci flow is shown in Fig. 4. With regularity term set to be 0.1, the Sinkhorn distance performs equally well as the optimal transport in Ricci flow process. And the time complexity is reduced by four times. The computation time is average over 5 iterations on an Intel(R) Xeon(R) CPU E5-2670 v2 @ 2.50GHz with 512G RAM using 20 processes. Optimal transport is computed by CVXPY(https://www.cvxpy.org) with ECOS solver, Sinkhorn distance is solved by POT: Python Optimal Transport(https://github.com/rflamary/POT).

4.4 Ricci flow with Surgery

Without too many parameter settings, Ricci flow yields good clustering performance for the graph with distinct community structures. In more complex graphs especially those with hierarchical community structures, we may need to run Ricci flow multiple times and do surgery during the Ricci flow iterations. For these kinds of graphs, we mimic the surgery process in the classic Ricci flow process during Ricci flow iterations. For every 5 iteration in the Ricci flow process, we cutoff the edges whose weights rank among the top 5%. Fig. 5 presents the edge weight and edge Ricci curvature changes during Ricci flow iterations with surgery. For every 5 iteration, the surgery process cuts out a small part of high weighted edges (mostly inter-community edges), and the surgery helps to further separate the communities.

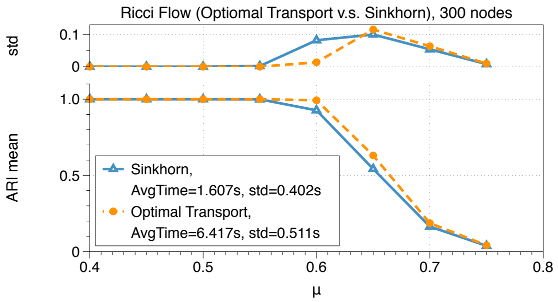

Figure 4. A comparison of applying Sinkhorn distance and optimal transport distance for Ricci flow computations on LFR graph with 300 nodes. The result of applying the Sinkhorn distance is similar as the one using optimal transport wile the time cost is four times smaller.

Fig. 6 demonstrates the result of Ricci flow with surgery. With LFR graph of 1000 nodes and $\mu = 0.6$, the surgery process boosts the ARI from 0.3 to 0.8.

For most of the networks that come without ground-truth community labels, we proposed to use modularity as an index to decide the final edge weight cutoff threshold to detect the communities. For a given graph, we first apply the graph with 20 to 50 iterations of discrete Ricci flow processes. (Iteration required varies by the complexity of the graph. For example, LFR with 1000 nodes in Fig. 5 takes around 50 iterations to fully stabilized.) Then we remove edges from highest to lowest, and log the modularity as Fig. 7. From the relationship of ARI and modularity we observed from Fig.6 in the main article, we suggest the cutoff threshold to be the point when modularity first hit the plateau of the curve. In Fig. 7 and Fig. 8, we represent three different models of GNet from planar to scale-free to demonstrate the final clustering results with different cutoff thresholds based on modularity. By properly adjusting the cutoff threshold, the hierarchical community structure of the graph is also revealed.

5 Proof of Theorem 4.1

We remark that we are not able to prove the similar result for other Ollivier-Ricci curvatures when $p > 0$ though numerical results indicate it should be true.

We start by computing the Wasserstein distance of a metric on $G(a,b)$. In each community C_i there is a specific node u_i which connects to other communities. We call this node the gateway node and the rest of the nodes in C_i the non-gateway nodes. There are three types of edges in the graph, edges connecting two communities (on two gateway nodes, such as u_1u_2 in Fig. 9), edges connecting a gateway node with a non-gateway node in the same community (such as u_2i and u_2j), and edges connecting two non-gateway nodes (such as ij).

Since the initial metric has edge length one and the Ricci flow preserves the graph symmetry, there are only three different edge lengths at each iteration of the Ricci flow, corresponding to the three types of edges. Suppose the edge lengths of the metric at the n-th iteration are d_1, d_2 and d_3 for the edges between communities, edges from a gateway node to a non-gateway node, and edges between two non-gateway nodes respectively, as shown in Fig. 9. Let edge lengths of the $(n+1)$-th iteration be D_1, D_2 and D_3 which are the Wasserstein distances of the corresponding edges for the metric graph $(G(a,b),d)$ with respect to the probability measures $\{\mu_x | x \in V\}$.

Lemma 5.1. *The Wasserstein distances D_1, D_2, D_3 are given by*

$D_1 = \frac{a-1}{a+b}d_1 + \frac{2a}{a+b}d_2,$

$D_2 = \frac{b}{a+b}d_1 + \frac{ab-a-b}{a(a+b)}d_2 + \frac{1}{a+b}d_3,$

$D_3 = \frac{1}{a}d_3.$

Furthermore, suppose $a > b \geq 2$ and $d_1 \geq d_2 \geq d_3$. Then $D_1 \geq D_2 \geq D_3$.

Proof: The easiest to compute is D_3. The vertices adjacent to i are the same as vertices adjacent to j. Furthermore, each vertex x adjacent to i (or j) carries the same mass $1/a$. See Fig. 9 (a). Thus the optimal transportation to move μ_i to μ_j is to transport the mass $1/a$ at j to i along the edge ij of distance d_3. Therefore, $D_3 = \frac{1}{a}d_3$ by definition. See Fig. 2 (a).

Now let us compute the Wasserstein distance D_1 of moving the probability measures μ_u to μ_v. Note that by definition the vertex degree $d_u = d_v = a+b$. Thus the probability measures μ_u and μ_v have mass $\frac{1}{a+b}$ at vertices adjacent to u or

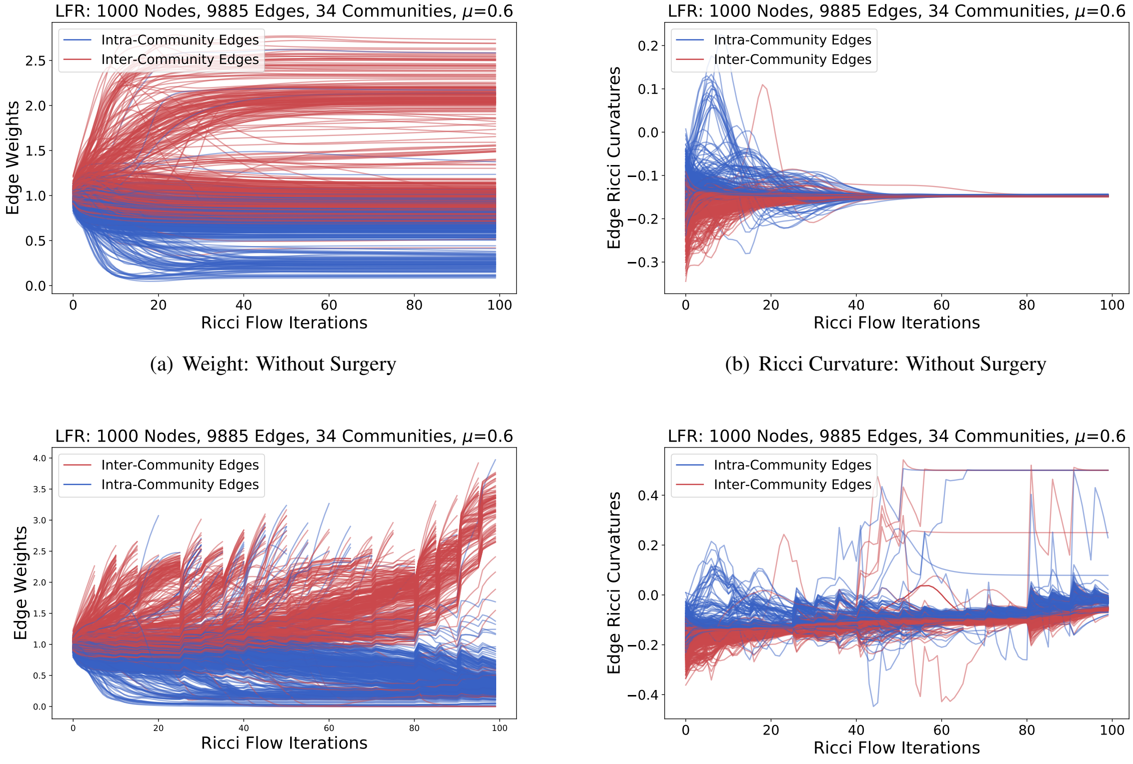

(a) Weight: Without Surgery

(b) Ricci Curvature: Without Surgery

(c) Weight: With Surgery

(d) Ricci Curvature: With Surgery

Figure 5. Edge weights and Ricci curvatures over Ricci flow iterations with and without surgery on a LFR graph with 1000 nodes, 9718 edges, and $\mu = 0.6$. In Fig. 5(c) and Fig. 5(d), for every 5 iterations, the surgery process cut out a small part of high weighted edges (mostly inter-community edges). This cutting helps further separate the communities.

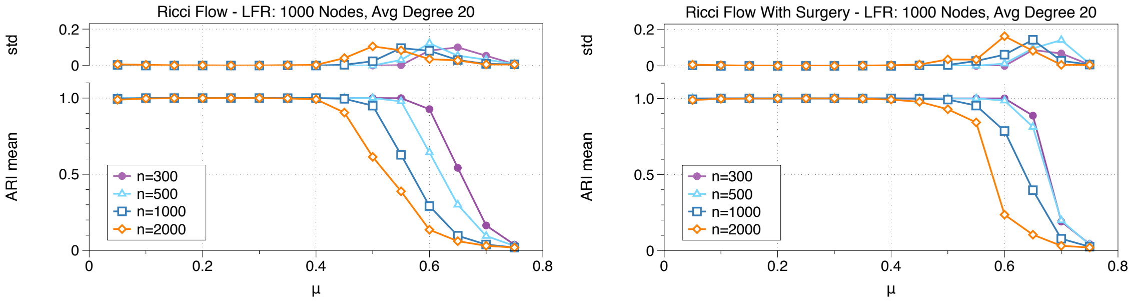

(a) Ricci Flow

(b) Ricci Flow With Surgery

Figure 6. Accuracy evaluation of 50 iterations of Ricci flow and 50 iterations Ricci flow with surgery for every 5 iterations. With LFR graph of 1000 nodes and $\mu = 0.6$, the surgery process boost the clustering accuracy ARI from 0.3 to 0.8.

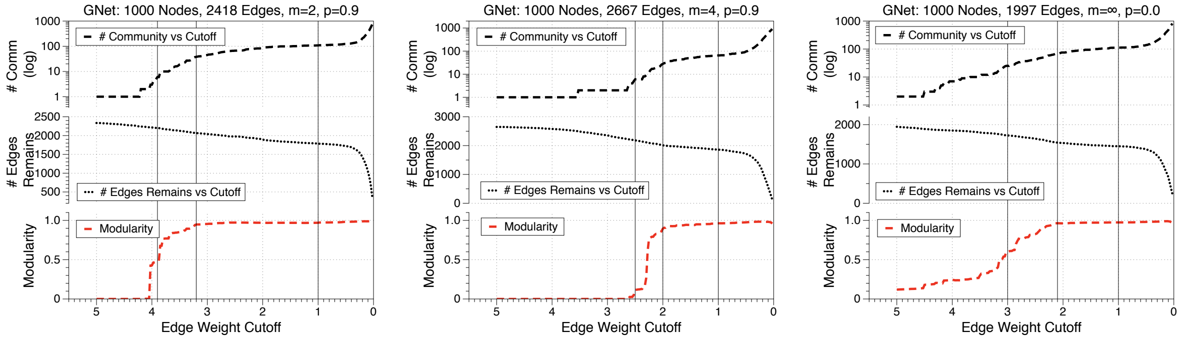

(a) GNet: $m = 2, p = 0.9$ (b) GNet: $m = 4, p = 0.9$ (c) GNet: $m = \infty, p = 0.0$

Figure 7. Modularity of GNet with 1000 nodes with different parameter setting. For each setting, we suggest the middle vertical line as cutoff threshold as it is the turning point for modularity curve. We also add two extra cutoff thresholds (left and right vertical line) for comparison. The result of communities detected with these three given cutoff thresholds are shown in Fig. 8.

v. There are $b-1$ vertices which are adjacent to both u and v such that each vertex has mass $\frac{1}{a+b}$ in both μ_u and μ_v. Therefore, there is no need to move them. We only need to move the mass $\frac{1}{a+b}$ at each of the rest a many vertices x adjacent to u to those vertices y adjacent to v and need to move the mass at v to the mass at u. The best transportation plan goes as follows.

- Step 1. Move the mass $\frac{1}{a+b}$ at vertex x to u along the edge xu. Since there are a many such vertices x, the total cost is $\frac{a}{a+b}d_2$ where d_2 is the distance from x to u.
- Step 2. Leave a mass of $\frac{1}{a+b}$ at u and move the rest of mass $\frac{a-1}{a+b}$ from u to v along uv. The total cost is $\frac{a-1}{a+b}d_1$.
- Step 3. Finally, move the mass $\frac{a}{a+b}$ at v to vertices y by equal distributions along edges vy of distance d_2. The total cost is $\frac{a}{a+b}d_2$.

Therefore, $D_1 = \frac{a-1}{a+b}d_1 + \frac{2a}{a+b}d_2$.

Finally, let us compute the Wasserstein distance D_2 of moving the measure μ_i to μ_u. The mass of μ_i at a vertex x adjacent to i is $\frac{1}{a}$ and the mass of μ_u at vertices adjacent to u is $\frac{1}{a+b}$. The best transportation plan is the following. Note that every vertex adjacent to i is also adjacent to u.

- Step 1. Leave the mass $\frac{1}{a+b}$ at each vertex $x \neq u$ adjacent to i. The total leftover mass at these vertices x is $\frac{(a-1)b}{a(a+b)}$. Move the mass of $\frac{1}{a+b}$ from the leftover mass at these x to the vertex i of distance d_3 from x. The total cost is $\frac{d_3}{a+b}$. After this move, there is a total mass of $\frac{(a-1)b}{a(a+b)} - \frac{1}{a+b} = \frac{ab-a-b}{a(a+b)}$ at these x. Move them to vertex u along edges of length d_2. The total cost is $\frac{ab-a-b}{a(a+b)}d_2$.
- Step 2. Finally, move a mass of $\frac{b}{a+b}$ at the vertex u to vertices y adjacent to u such that y is not adjacent to i. The total cost is $\frac{bd_1}{a+b}$.

Therefore, the cost of transportation is $D_2 = \frac{d_3}{a+b} + \frac{ab-a-b}{a(a+b)}d_2 + \frac{bd_1}{a+b}$.

Now we come to prove the last statement. Since $d_1 \geq d_2 \geq d_3$, we obtain $D_2 \geq \frac{b}{a+b}d_1 \geq \frac{1}{a}d_1 \geq \frac{1}{a}d_3 = D_3$. Also,

$$
\begin{aligned}
D_1 - D_2 &= \frac{a(a-b-1)}{a(a+b)}d_1 + \frac{2a^2-ab+a+b}{a(a+b)}d_2 - \frac{a}{a(a+b)}d_3 \\
&\geq \frac{d_2}{a(a+b)}[a(a-b-1)+2a^2-ab+a+b-a] \\
&= \frac{d_2}{a(a+b)}[a(a-b-1)+a^2-ab+a^2+b] \geq 0.
\end{aligned}
$$

□

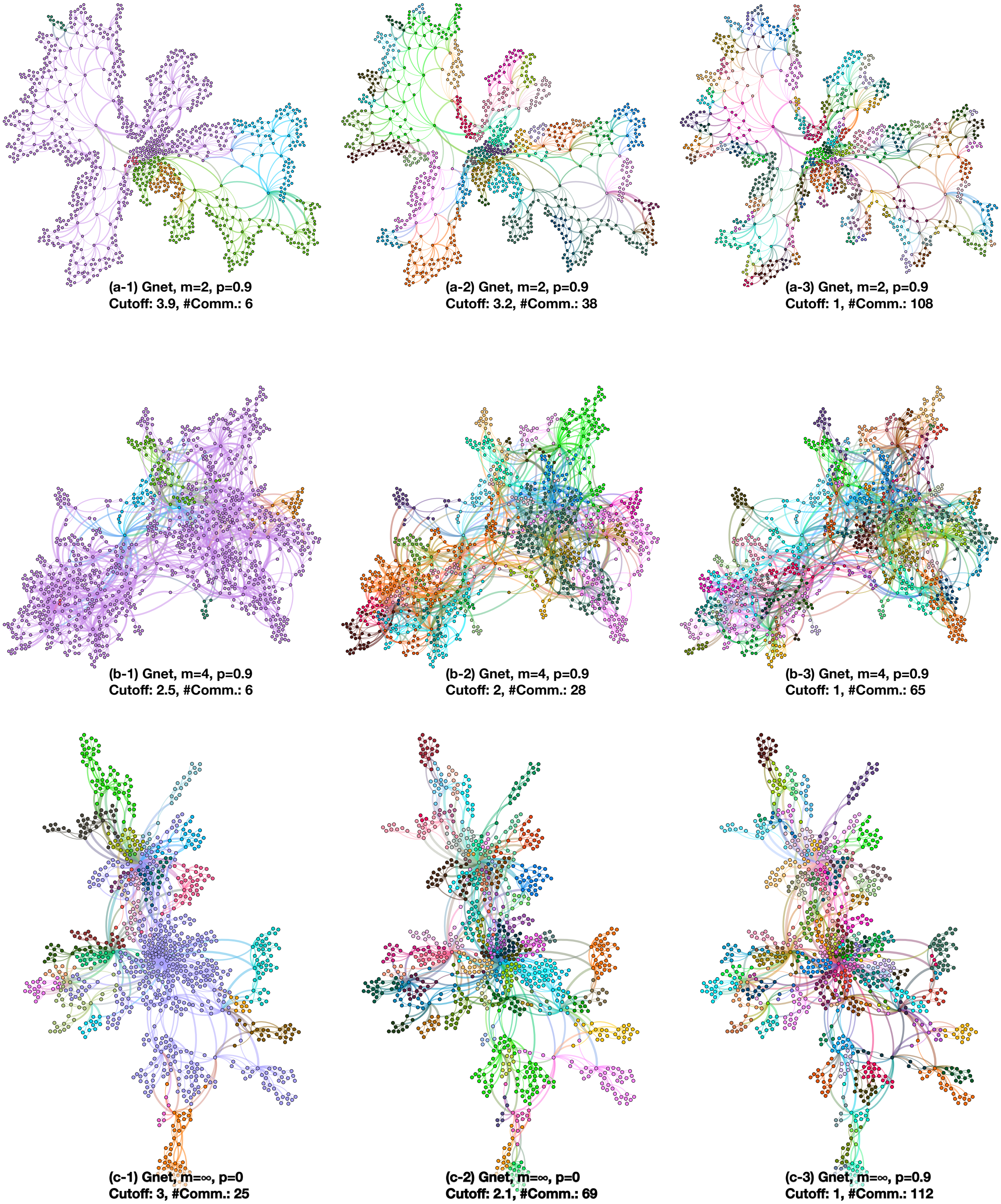

Figure 8. The emergent geometrical network model generates the following networks:7(a) a network with random planar geometry for $m = 2, p = 0.9$; 7(b) a network with a broad degree distribution, small-world property, and finite spectral dimension for $m = 4, p = 0.9$; 7(c) and a scale-free network with power-law degree distribution for $m = \infty, p = 0.9$.

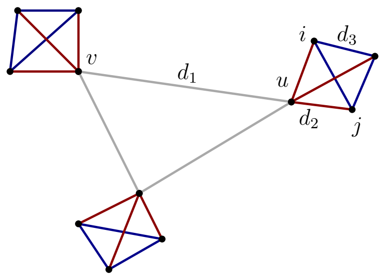

Figure 9. The graph $G(a,b)$ obtained from a complete graph on $b+1$ vertices by replacing each vertex by a complete graph of $a+1$ vertices. In this figure, $a=3$, $b=2$.

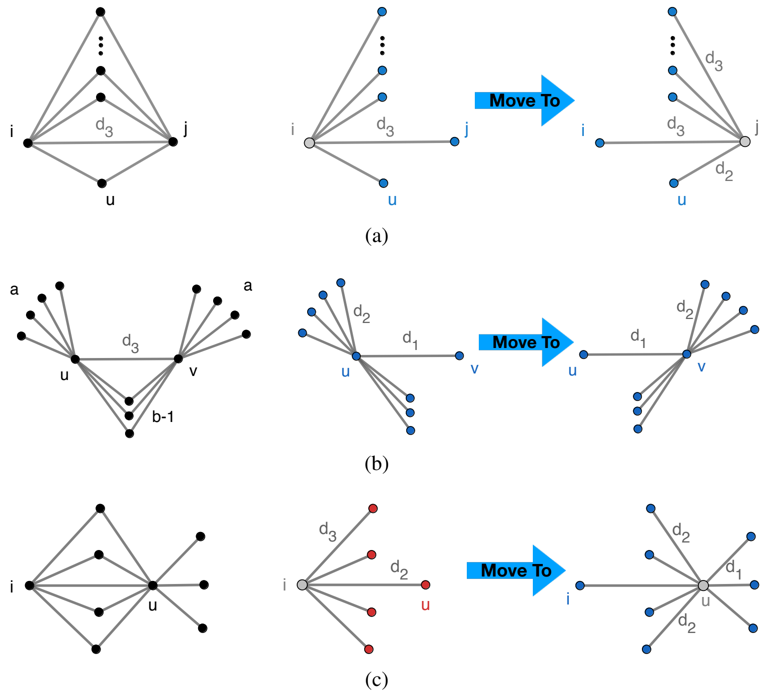

Figure 10. Parts (a), (b) and (c) illustrate the optimal transportation to move the mass at vertex u to vertex v.

Let A be the 3×3 matrix

$$\begin{bmatrix} \frac{a-1}{a+b} & \frac{2a}{a+b} & 0 \\ \frac{b}{a+b} & \frac{ab-a-b}{a(a+b)} & \frac{1}{a+b} \\ 0 & 0 & \frac{1}{a} \end{bmatrix}$$

and $W_n = [w_{n1}, w_{n2}, w_{n3}]^t$ be the 3×1 column vector given by $W_{n+1} = AW_n$ with $W_0 = [1,1,1]^t$. Then w_{ni} are the edge lengths of the graph $G(a,b)$ after n-th iteration of the Ricci flow. Our goal is the understand the asymptotic behavior of W_n.

Using Maple calculation, we conclude the following lemma.

Lemma 5.2. *Suppose $a > b \geq 2$, then there are three real eigenvalues $\lambda_1 > \lambda_2 = \frac{1}{a} \geq 0 > \lambda_3$ of the matrix A. The largest eigenvalue $\lambda_1 > \lambda_3$. Furthermore, an eigenvector w_1 associated to λ_1 is of the form $[1,k,0]^t$ where k is in the open interval $(0,1)$.*

By this lemma, we conclude the proof of the theorem as follows. Let w_2 and w_3 be the eigenvectors associated to λ_1 and λ_3 of A. The $\lambda_2 = \frac{1}{a}$ eigenvector $w_2 = [0,0,1]^t$. Since $\lambda_1, \lambda_2, \lambda_3$ are distinct, the eigenvectors w_1, w_2, w_3 are linearly independent in $\mathbb{R}^3$.

Write the vector $W_0 = [1,1,1]^t = a_1 w_1 + a_2 w_2 + a_3 w_3$. Then using $w_1 = [1,k,0]^t$ and $\lambda_1 > |\lambda_2|, |\lambda_3|$, we obtain

$$W_n = a_1 \lambda_1^n w_1 + a_2 \lambda_2^n w_2 + a_3 \lambda_3^n w_3 = \begin{bmatrix} a_1 \lambda_1^n + o(\lambda_1^n) \\ k a_1 \lambda_1^n + o(\lambda_1^n) \\ (\frac{1}{a})^n \end{bmatrix}.$$

Here $o(\lambda_1^n)$ stands for an expression such that $\lim_{n\to\infty} o(\lambda_1^n)/\lambda_1^n = 0$. As a conclusion, we see that the distance at the edge uv grows at the rate of λ_1^n, the distance at the edges ui and ij grows at rate $o(\lambda_1^n)$. In fact, the distance the edge ij grows at the rate of $\frac{1}{a} < 1$ and shrinks to zero exponentially fast.

References

1. Kantorovich, L. V. On the translocation of masses. In *Dokl. Akad. Nauk. USSR (NS)*, vol. 37, 199–201 (1942).
2. Ollivier, Y. Ricci curvature of markov chains on metric spaces. *J. Funct. Anal.* **256**, 810–864 (2009).
3. Lin, Y., Lu, L. & Yau, S.-T. Ricci curvature of graphs. *Tohoku Math. J.* **63**, 605–627 (2011).
4. Chow, B., Luo, F. *et al.* Combinatorial Ricci flows on surfaces. *J. Differ. Geom.* **63**, 97–129 (2003).
5. Girvan, M. & Newman, M. E. J. Community structure in social and biological networks. *Proc. Natl. Acad. Sci. U. S. A.* **99**, 7821–7826 (2002).
6. Ni, C.-C., Lin, Y.-Y., Gao, J., Gu, X. D. & Saucan, E. Ricci curvature of the internet topology. In *IEEE. Ic. Comp. Com. Net. (INFOCOM)*, vol. 26, 2758–2766 (IEEE, 2015). https://doi.org/10.1109/INFOCOM.2015.7218668.
7. Samal, A. *et al.* Comparative analysis of two discretizations of Ricci curvature for complex networks. *Sci. Rep.* **8**, 8650 (2018).
8. Saucan, E., Samal, A., Weber, M. & Jost, J. Discrete curvatures and network analysis. *MATCH Commun. Math. Comput. Chem.* **80**, 605–622 (2018).
9. Sreejith, R. P., Jost, J., Saucan, E. & Samal, A. Systematic evaluation of a new combinatorial curvature for complex networks. *Chaos Solitons Fractals* **101**, 50–67 (2017).
10. Jost, J. & Liu, S. Ollivier's Ricci curvature, local clustering and Curvature-Dimension inequalities on graphs. *Discret. Comput. Geom.* **51**, 300–322 (2014).
11. Milnor, J. A unique decomposition theorem for 3-manifolds. *Amer. J. Math.* **84**, 1–7 (1962).
12. Jaco, W. & Shalen, P. B. SEIFERT FIBERED SPACES IN 3-MANIFOLDS. In Cantrell, J. C. (ed.) *Geometric Topology*, 91–99 (Academic Press, 1979).
13. Johannson, K. *Homotopy Equivalences of 3-Manifolds with Boundaries* (Springer, 2006).
14. Thurston, W. P. Three dimensional manifolds, kleinian groups and hyperbolic geometry. *Bull. Am. Math. Soc.* **6**, 357–382 (1982).
15. Hamilton, R. S. Three-manifolds with positive ricci curvature. *J. Differ. Geom.* **17**, 255–306 (1982).
16. Morgan, G., John; Tian. Ricci flow and the poincaré conjecture. *Clay Math. Monogr.* **3**, 521 (2007).
17. Spanier, E. H. Algebraic topology. (1966).
18. Cuturi, M. Sinkhorn distances: Lightspeed computation of optimal transport. In Burges, C. J. C., Bottou, L., Welling, M., Ghahramani, Z. & Weinberger, K. Q. (eds.) *Advances in Neural Information Processing Systems 26*, 2292–2300 (Curran Associates, Inc., 2013).
19. Hubert, L. & Arabie, P. Comparing partitions. *J. Classif.* **2**, 193–218 (1985).
20. Rand, W. M. Objective criteria for the evaluation of clustering methods. *J. Am. Stat. association* **66**, 846–850 (1971).
21. Newman, M. E. & Girvan, M. Finding and evaluating community structure in networks. *Phys. Rev. E* **69**, 026113 (2004).
22. Fortunato, S. & Barthélemy, M. Resolution limit in community detection. *Proc. Natl. Acad. Sci. U. S. A.* **104**, 36–41 (2007).
